# Effect of Grain-Boundary Discontinuous Precipitation Evolution on the Transition of Creep-Rupture Failure Modes in Ni–Fe-Based Alloy/Inconel 617 Dissimilar Welded Joints

**DOI:** 10.3390/ma19183859

**Published:** 2026-09-10

**Authors:** Linshu Li, Shengzhi Li, Manjie Fan, Jun Cheng, Wuhua Zhang, Xin Huo, Xia Liu, Kejian Li, Zhipeng Cai, Qu Liu

**Affiliations:** 1 Department of Mechanical Engineering, Tsinghua University, Beijing 100084, China; li-ls24@mails.tsinghua.edu.cn (L.L.); chengj22@mails.tsinghua.edu.cn (J.C.); zhangwh22@mails.tsinghua.edu.cn (W.Z.); kejianli@mail.tsinghua.edu.cn (K.L.); czpdme@mail.tsinghua.edu.cn (Z.C.); 2 Shanghai Turbine Plant, Shanghai Electric Power Generation Equipment Co., Ltd., Shanghai 200240, China; lishzh@shanghai-electric.com (S.L.); fanmj@shanghai-electric.com (M.F.); huoxin@shanghai-electric.com (X.H.); liuxia@shanghai-electric.com (X.L.); 3 State Key Laboratory of Tribology in Advanced Equipment, Tsinghua University, Beijing 100084, China; 4 Key Laboratory for Advanced Materials Processing Technology, Ministry of Education, Beijing 100084, China

**Keywords:** Ni–Fe-based alloy, dissimilar welded joint, discontinuous precipitation, creep rupture, grain-boundary stability

## Abstract

The creep-rupture behavior and microstructural evolution of Ni–Fe-based alloy/Inconel 617 dissimilar welded joints were investigated by creep-rupture testing, interrupted creep testing, and microstructural characterization. Tests were conducted at 630–750 °C under stresses of 130–375 MPa. All joint specimens fractured in the heat-affected zone (HAZ) or base metal (BM) on the Ni–Fe-based alloy side, indicating that the Ni–Fe-based alloy side was the creep-critical region of the joint. Three failure regimes were identified according to the relative rupture lives of the joints and the Ni–Fe-based alloy BM. Fractographic observations revealed predominantly intergranular fracture, with creep cavities and cracks preferentially associated with grain-boundary discontinuous precipitation (DP) regions containing coarsened rod-like γ′ precipitates and precipitate-free zones (PFZs). Differences in DP evolution between the HAZ and BM were closely related to creep-damage localization and failure behavior. Interrupted creep tests showed that temperature was the dominant factor affecting DP formation and growth, while applied stress accelerated its evolution. DP developed rapidly during the initial exposure stage and subsequently grew more slowly. These findings clarify the relationship between grain-boundary DP evolution and creep-rupture failure in Ni–Fe-based alloy/Inconel 617 welded joints, and also provide microstructural guidance for assessing and mitigating creep degradation in dissimilar welded components used in high-temperature A-USC systems. Future work should combine longer-term creep testing, phase-resolved microstructural characterization, and predictive modeling to establish quantitative relationships among DP evolution, creep-damage accumulation, and rupture life.

## 1. Introduction

With the rapid development of China’s economy and industry, electricity demand continues to increase [1,2,3]. Advanced ultra-supercritical (A-USC) coal-fired power generation, which further increases steam parameters, can substantially improve power-generation efficiency and reduce CO_2_ emissions and is an important route toward efficient and cleaner coal utilization [4,5]. However, service temperatures of 650 °C and above impose increasingly stringent requirements on materials used for high-temperature components, and conventional heat-resistant steels cannot satisfy the demands of long-term service at elevated temperatures [6]. Therefore, the development of new superalloys combining excellent high-temperature strength with microstructural stability is essential for advancing A-USC technology [7].

Nickel-based superalloys are widely regarded as promising candidate materials for critical high-temperature components in A-USC power plants because of their excellent high-temperature mechanical properties, oxidation resistance, and corrosion resistance [8,9]. Nickel-based alloys such as Inconel 740H and 617B have been used in related research and demonstration projects, but their high cost and limited processability restrict large-scale engineering applications [10]. To meet the requirements of large A-USC components, a new Ni–Fe-based alloy was jointly developed by Xi’an Thermal Power Research Institute Co., Ltd. (Xi’an, China) and China Huaneng Group Co., Ltd. (Beijing, China). By increasing the Fe content and reducing the Co content, this alloy combines high-temperature performance with improved economy and processability and has considerable engineering potential.

For γ′-strengthened nickel-based superalloys, grain-boundary stability is a major factor governing high-temperature service performance. Under the combined effects of temperature and stress, grain boundaries can migrate and undergo grain-boundary discontinuous precipitation (DP) and coarsening, producing DP regions composed of abnormally coarsened rod-like γ′ precipitates and precipitate-free zones (PFZs) [11,12]. Previous studies have shown that grain-boundary DP regions mainly develop during the later stages of creep and are closely associated with grain-boundary migration [13,14,15,16]. Their formation and evolution are affected by temperature, stress state, microstructural characteristics, and grain-boundary carbides [17,18,19,20].

The formation of grain-boundary DP regions not only indicates a loss of grain-boundary microstructural stability but also significantly degrades high-temperature performance. Grain-boundary DP regions have been reported to promote creep-cavity nucleation and crack propagation, thereby accelerating material failure [21,22]. They can also reduce the yield strength of an alloy, with the magnitude of the reduction being closely related to the size of the DP regions [23]. Therefore, grain-boundary DP is an important microstructural indicator for evaluating grain-boundary stability and high-temperature reliability in nickel-based superalloys.

Nevertheless, the grain-boundary DP behavior of the Ni–Fe-based alloy and its effect on the creep-rupture failure of dissimilar welded joints have not been systematically investigated. Although grain-boundary DP has been extensively investigated in commercial nickel-based superalloys such as Inconel 740H and Nimonic alloys, relevant studies on the newly developed Ni–Fe-based alloy are still limited. In particular, the formation conditions and microstructural evolution of grain-boundary DP in the Ni–Fe-based alloy, as well as its influence on creep-rupture failure, have not been clarified. Moreover, despite the widespread use of dissimilar welded structures in critical A-USC components, the high-temperature service performance and failure mechanisms of Ni–Fe-based alloy/Inconel 617 dissimilar welded joints have not been systematically investigated. In this work, Ni–Fe-based alloy/Inconel 617 dissimilar welded joints were subjected to creep-rupture tests, interrupted creep tests, and microstructural characterization to investigate their failure behavior under different stress conditions and to clarify the role of grain-boundary DP in joint failure. The effects of temperature, stress, and time on the formation and evolution of grain-boundary DP in the Ni–Fe-based alloy were further studied to establish its formation conditions and evolution characteristics. The results provide a theoretical basis for optimizing welding and heat-treatment procedures and improving the high-temperature reliability of Ni–Fe-based alloy dissimilar welded joints.

## 2. Materials and Methods

### 2.1. Materials

The base materials were a Ni–Fe-based alloy and Ni-based Inconel 617, and ERNiCrCoMo-1 wire was used as the filler metal. Their chemical compositions are listed in Table 1. After forging, the materials were solution-treated at 980 °C for 1 h, and water was used to quench them to room temperature.

**Figure 1:**
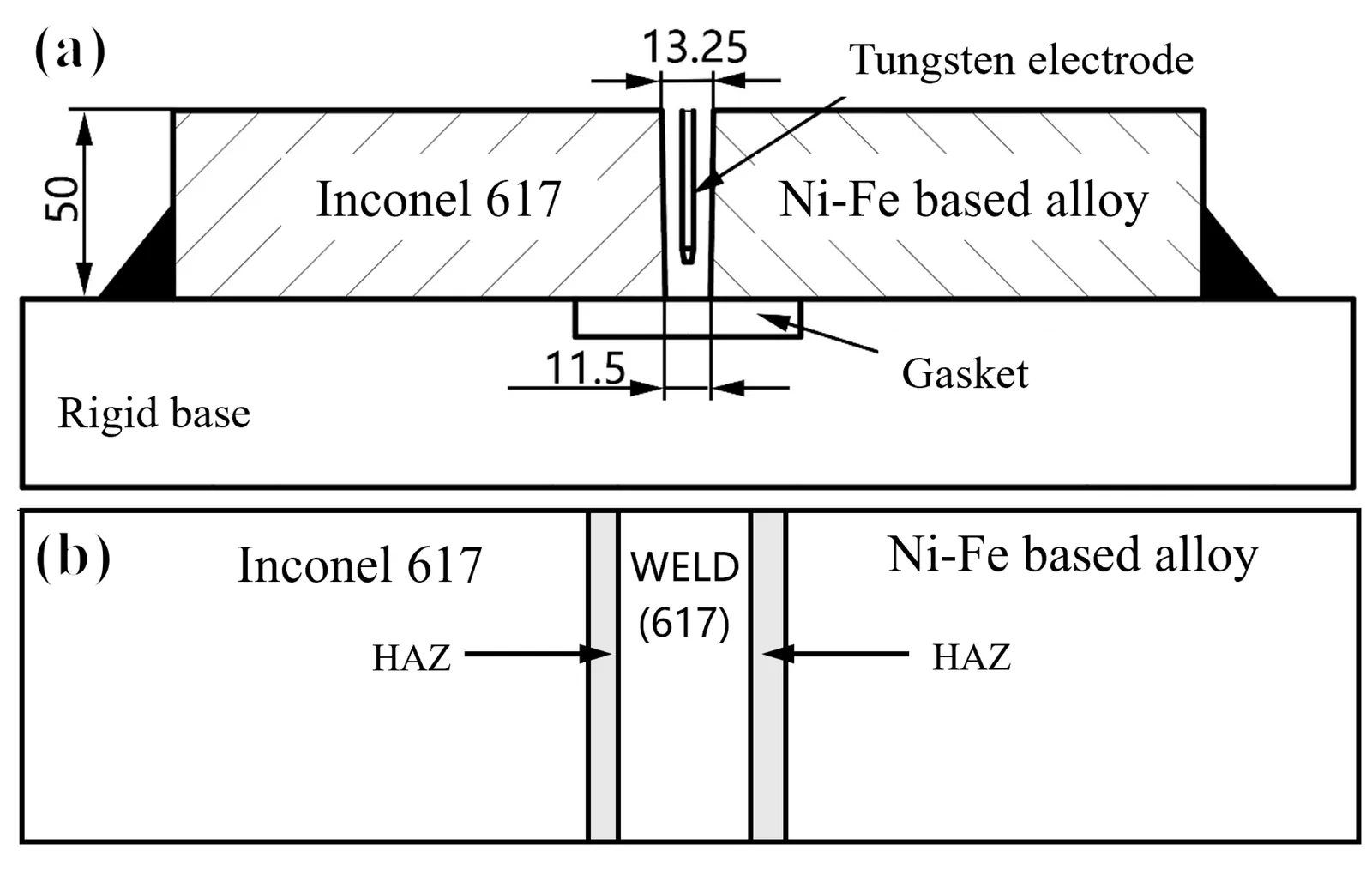
Welding procedure and joint configuration: (**a**) welding procedure; (**b**) welded-joint configuration.

The joint was fabricated by butt welding Inconel 617 and Ni–Fe-based alloy plates, each measuring 400 mm × 130 mm × 50 mm. Single-pass-per-layer, multi-layer narrow-gap tungsten inert gas (TIG) welding was employed. The welding procedure and joint configuration are shown in Figure 1. The welded joint was subjected to post-weld heat treatment at 980 °C for 1 h followed by water quenching to room temperature, and then aged at 650 °C for 2 h and 800 °C for 2 h. After satisfactory nondestructive inspection, the joint was sectioned perpendicular to the weld into several plates for subsequent creep-rupture testing and microstructural characterization.

### 2.2. Creep-Rupture Tests

**Figure 2:**
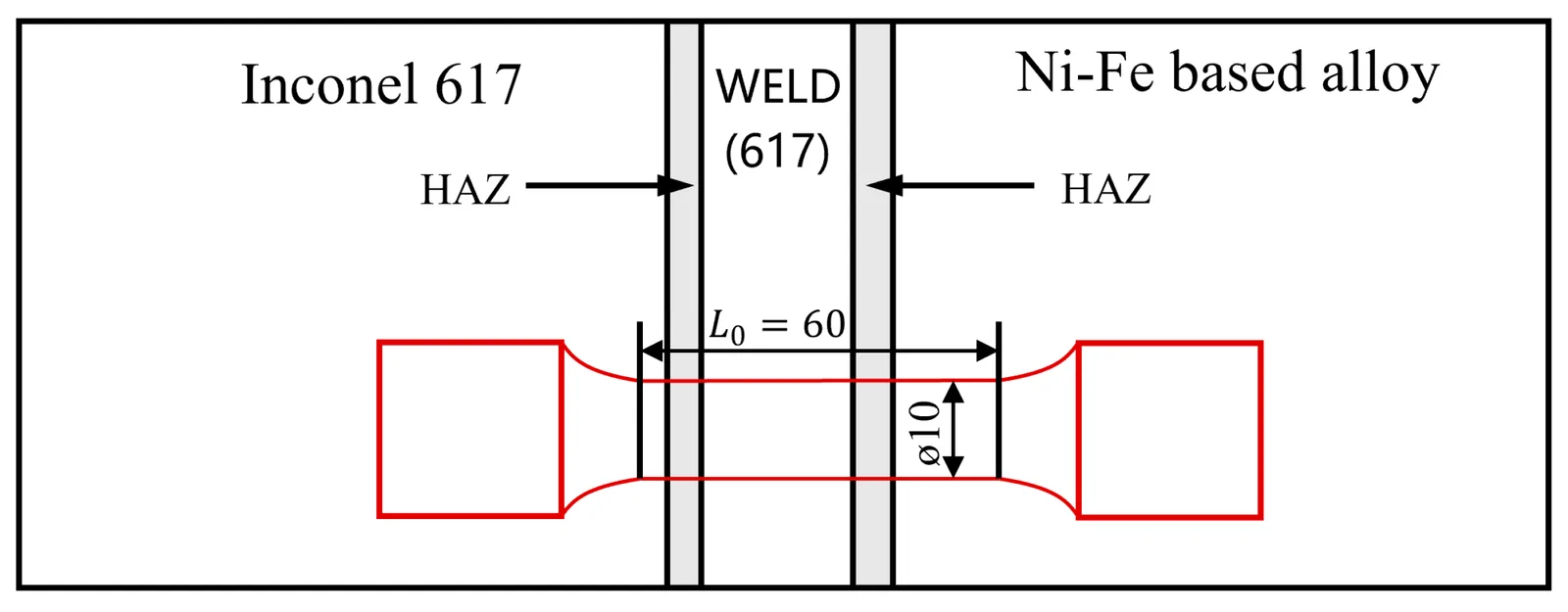
Schematic illustration of creep-rupture specimen extraction from the welded joint.

Creep-rupture tests of the Ni–Fe-based alloy joints were conducted in accordance with the GB/T 2039-2024, Metallic Materials—Uniaxial Creep Testing Method in Tension [24]. The welded joints and standard heat-treated Ni–Fe-based alloy were tested using cylindrical specimens with a diameter of 10 mm and an initial gauge length $L_{0}$ of 60 mm. The specimens were cut by wire electrical discharge machining perpendicular to the welding direction, with the weld located at the center of the gauge section and the HAZ and BM positioned on either side, as illustrated in Figure 2. Creep-rupture tests were performed over a broad range of service-relevant conditions, with temperatures of 630–750 °C and applied stresses of 130–375 MPa. The specimen temperature was monitored using S-type thermocouples, and the furnace temperature was maintained within ±1 °C of the nominal test temperature throughout the creep-rupture tests. A constant load was maintained using an RDL100 creep-rupture testing machine (Sinotest Equipment Co., Ltd., Changchun, China).

### 2.3. Microstructural Characterization

After creep-rupture testing, the specimens were sectioned by wire electrical discharge machining, sequentially ground using SiC abrasive papers, mechanically polished, and etched at room temperature for approximately 7 min using a solution consisting of 30 mL glycerol, 25 mL hydrochloric acid, and 5 mL nitric acid. The microstructures were examined using an optical microscope (OM; OLYMPUS DP72, Olympus Corporation, Tokyo, Japan) and a scanning electron microscope (SEM; GeminiSEM 300, Carl Zeiss Microscopy GmbH, Oberkochen, Germany). Electron probe microanalysis (EPMA; JXA-8230, JEOL Ltd., Tokyo, Japan) was employed for local chemical-composition analysis. The EPMA measurements were performed at an accelerating voltage of 15 kV and a beam current of 20 nA.

For phase identification of the rod-like precipitates within the grain-boundary discontinuous precipitation (DP) regions, site-specific thin foils were prepared by focused ion beam (FIB; Helios 5CX, FEI Company, Hillsboro, OR, USA) milling. The foils were thinned to approximately 80 nm before transmission electron microscopy (TEM) examination. A transmission electron microscope (Tecnai G2 F20 S-TWIN, FEI Company, Hillsboro, OR, USA) was used for bright-field imaging, selected-area electron diffraction (SAED), and energy-dispersive X-ray spectroscopy (EDS) analysis. The combined SAED and EDS results were used to determine the crystallographic and compositional characteristics of the rod-like precipitates.

### 2.4. Interrupted Creep Tests

Interrupted creep tests were conducted to capture the temporal evolution of grain-boundary DP and to establish the relationship between DP evolution and creep degradation. A solution-treated Ni–Fe-based alloy was used to isolate the effects of temperature, stress, and exposure time and to eliminate pre-existing grain-boundary DP introduced by the welding thermal cycle. The test temperatures ranged from 650 to 800 °C, and the holding times were 0.1, 0.2, 0.5, 1, 2, 5, 10, and 20 h. At 650 °C, applied stresses of 70–350 MPa were used. The finite-element analysis described in Section 2.5 indicated that the representative residual-stress level in the Ni–Fe-based alloy-side HAZ after solution treatment was approximately 150 MPa; therefore, 150 MPa was selected as the representative test stress at 700–800 °C. The interrupted creep tests were also conducted according to GB/T 2039-2024 using the same equipment as that described in Section 2.2. Because the diameter of the grip section was approximately twice that of the gauge section, the corresponding nominal stress in the grip section was approximately 37.5 MPa. The gauge and grip sections were therefore used as high- and low-stress regions, respectively. For comparison, previously obtained zero-stress isothermal-aging results at 700 and 750 °C and a zero-stress control condition at 800 °C were also considered. After 20 h of exposure under different temperature and stress conditions, the specimens were first examined at an SEM magnification of 500× to identify obvious grain-boundary DP. Specimens showing little or no apparent DP during the initial screening were subsequently examined at 3000×. For each specimen, 50 non-overlapping SEM fields of view at 3000× were systematically examined. A specimen was classified as exhibiting little or no apparent DP only when DP regions were absent or only sparsely observed across these fields. The same 50 micrographs were subsequently used for quantitative analysis of the grain-boundary DP fraction, mean DP-region length, and mean DP-region width.

**Figure 3:**
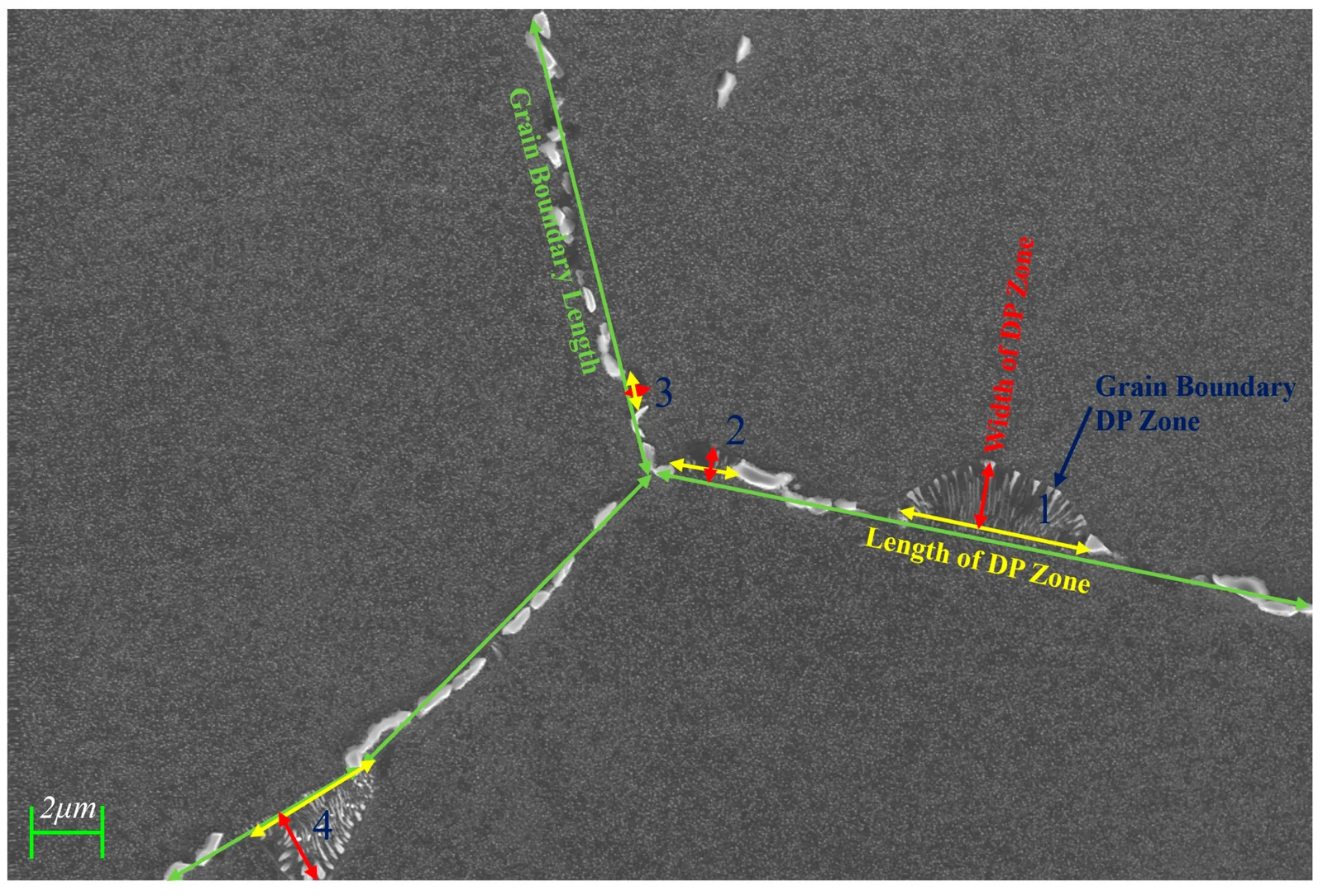
Schematic definitions of the three parameters used to quantify grain-boundary DP.

To quantitatively characterize the evolution of grain-boundary DP in a Ni–Fe-based alloy, statistical analyses were performed for each test condition. Triple-junction regions were selected as the principal observation sites, and 50 SEM micrographs at a magnification of 3000× were acquired for each specimen. The quantitative measurements were performed using a custom program developed in Python (version 3.11.5) developed for this study. For each micrograph, the target grain-boundary DP regions were manually identified, and the relevant geometric features, including DP-region length and width, were selected by the operator. The program subsequently calculated the grain-boundary DP fraction, mean DP-region length, and mean DP-region width. The SEM fields used for quantitative analysis were acquired independently of the apparent severity of DP to minimize operator-selection bias. Their definitions are illustrated in Figure 3.

**Figure 4:**
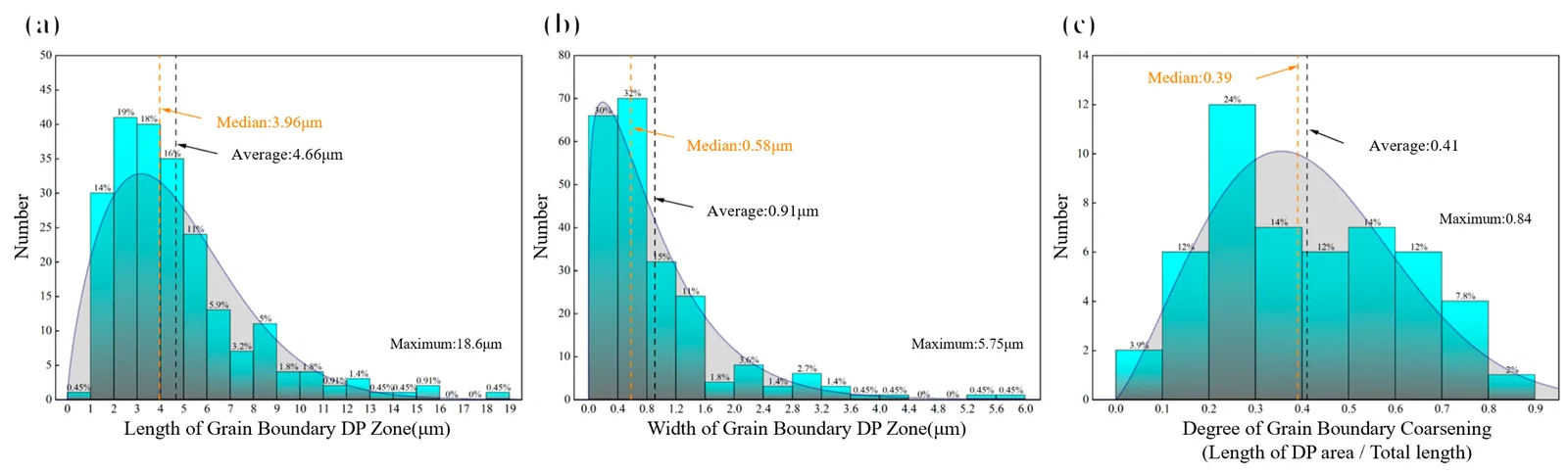
Weibull statistical analysis of the three characteristic parameters of grain-boundary DP: (**a**) DP-region length; (**b**) DP-region width; and (**c**) degree of grain-boundary coarsening. The histogram bars represent the experimental frequency distributions, the continuous curves represent the fitted Weibull probability density functions (PDFs), and the shaded regions indicate the areas under the corresponding fitted PDF curves.

Because grain-boundary DP exhibits intrinsic randomness and spatial heterogeneity, the probability distributions of the three parameters were analyzed and fitted using Weibull distribution functions, as shown in Figure 4. The mean values obtained from the fits were used as quantitative indices for evaluating the effects of temperature, stress, and time on grain-boundary DP evolution.

### 2.5. Finite Element Simulation of Post-Weld Residual Stress

A finite element model of a Ni–Fe-based alloy/Inconel 617 ring-welded joint was developed in ABAQUS 2022 to estimate the residual-stress scale after welding and post-weld heat treatment. A coupled temperature–displacement analysis was used. The mesh was refined in the weld region and gradually coarsened away from the weld to improve computational efficiency. The final model contained 11,483 nodes and 11,334 elements, with a minimum element size of 1 mm × 1 mm. Because the simulated ring geometry differed from the flat butt-welded plate used for creep testing, the calculated stresses were used as a reference for selecting interrupted-test conditions rather than as a direct prediction of the residual-stress field in the creep specimens.

In the finite element analysis of the multi-layer, multi-pass weld, the element birth-and-death technique was used to model deposition of each weld pass. All weld elements were initially deactivated and were subsequently reactivated during the simulated welding process. To improve computational efficiency, two adjacent physical passes were combined into one simulated pass, reducing the welding process to 108 coupled heating and cooling analysis steps. The initial preheating temperature was 80 °C. After each pass, the joint was cooled to 130 °C before deposition of the next pass. After the final pass, the entire joint was cooled to room temperature before the residual stress was evaluated.

Thermal and mechanical fields were calculated simultaneously. The transient nodal temperature field provided the thermal loading for the mechanical analysis. Solid-state phase transformation was neglected because neither the Inconel 617 nor the Ni–Fe-based alloy was assumed to undergo such transformation under the modeled conditions. Creep strain during the short local high-temperature welding cycle was also neglected. The total strain was expressed as the sum of elastic, plastic, and thermal components:

(1) $$\varepsilon_{total}=\varepsilon_{e}+\varepsilon_{p}+\varepsilon_{th}$$

where $\varepsilon_{e}$, $\varepsilon_{p}$, and $\varepsilon_{th}$ denote the elastic, plastic, and thermal strain components, respectively. The elastic strain was calculated according to Hooke’s law using temperature-dependent material properties such as Young’s modulus and Poisson’s ratio. The thermal strain was expressed as follows:

(2) $$\varepsilon_{th}=\alpha_{T}\cdot \Delta T$$

where $\alpha_{T}$ is the coefficient of thermal expansion at temperature T. According to Equation (2), thermal strain drives overall deformation compatibility in the welded joint. When the local stress exceeds the temperature-dependent yield strength, plastic strain develops, causing strain hardening and changes in the yield limit and thereby affecting subsequent plastic deformation. The thermo-elastoplastic stress calculation adopted the Von Mises yield criterion, and isotropic hardening was used in the plastic regime to describe strain hardening.

After completion of the welding simulation and cooling to room temperature, the calculated as-welded residual-stress field was retained as the initial state for the subsequent solution-treatment simulation. A coupled temperature–displacement analysis was employed. The model was heated to 980 °C, held at this temperature for 1 h, and subsequently cooled to room temperature. During the solution-treatment cycle, convective and radiative heat transfer were applied to the external surfaces. The ambient temperature was set to 20 °C, with a convective heat-transfer coefficient of 20 W m^−2^ K^−1^ and a surface emissivity of 0.8. The mechanical boundary conditions were maintained consistently with those used during the welding simulation: axisymmetric constraints were imposed, and one corner point was fixed to suppress rigid-body motion while allowing thermal expansion and contraction. Temperature-dependent thermophysical and mechanical properties were employed throughout the thermal cycle.

## 3. Results and Discussion

### 3.1. Creep-Rupture Properties of the Joints

**Figure 5:**
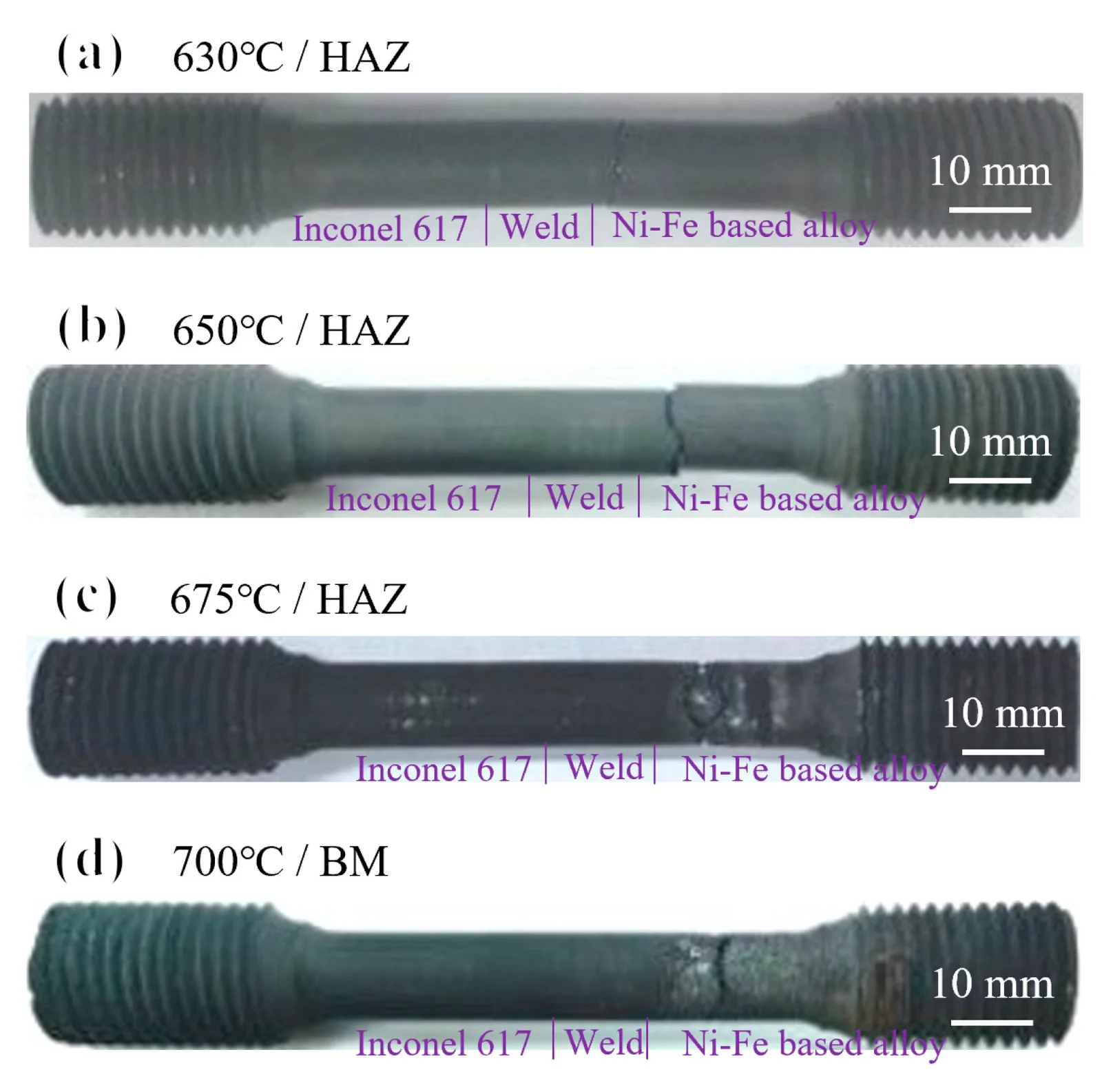
Macroscopic appearances of the creep-ruptured Ni–Fe-based alloy joint specimens.

Representative macroscopic appearances of the creep-ruptured Ni–Fe-based alloy joint specimens are shown in Figure 5. All specimens failed in the Ni–Fe-based alloy-side HAZ or BM. Compared with the solution-strengthened weld metal, the precipitation-strengthened Ni–Fe-based alloy side was the creep-critical region of the joint. The elongation after rupture and reduction in area were low for all specimens, and no pronounced macroscopic elongation or necking was observed, indicating predominantly brittle creep-rupture behavior.

In addition to the welded-joint tests, creep-rupture tests were performed over a broad range of service-relevant conditions on standard heat-treated Ni–Fe-based alloy base metal.

The Larson–Miller parameter (LMP) was used to correlate the creep-rupture data obtained at different temperatures. The LMP combines absolute temperature, T, and rupture time, $t_{r}$ as follows:

(3) $$LMP=T\left(C+logt_{r}\right)$$

where *C* is the Larson–Miller constant and depends on the material; *C* = 20, a value commonly used for nickel-based superalloys and heat-resistant steels, was adopted in this study. The LMP was then linearly fitted against the logarithm of stress σ, as follows:

(4) $$LMP=a_{0}+a_{1}log\sigma$$

where $a_{0},a_{1}$ are the fitting coefficients.

**Figure 6:**
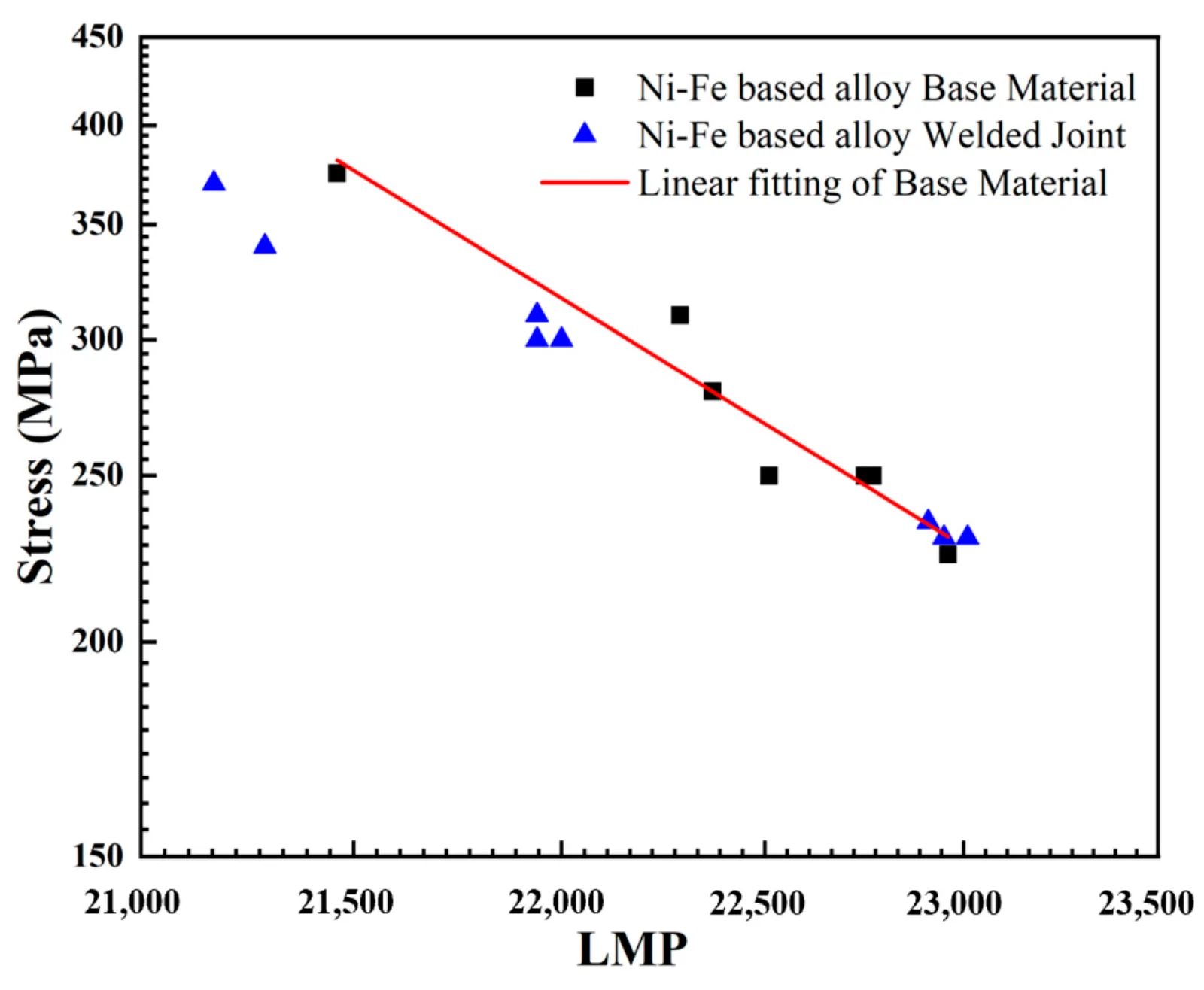
Creep-rupture data of the Ni–Fe-based alloy base metal and welded joints.

The creep-rupture data for the welded joints and Ni–Fe-based alloy BM were plotted in Larson–Miller form, as shown in Figure 6. The BM data were used to provide a reference trend for comparing the welded-joint results.

Figure 6 shows that the difference in creep-rupture performance between the welded joint and the Ni–Fe-based alloy base metal was strongly stress-dependent. As the applied stress decreased, the difference in creep-rupture life gradually diminished. Based on the relative creep-rupture performance of the joint and base metal, the failure behavior was classified into three characteristic modes:

Mode I (Comparable-Life Regime): The joint and BM showed broadly comparable creep-rupture lives;

Mode II (Moderately Reduced-Life Regime): The joint showed a moderately shorter creep-rupture life than the BM;

Mode III (Markedly Reduced-Life Regime): The joint showed a substantially shorter creep-rupture life than the BM.

The three regimes were classified according to the relative positions of the welded-joint data points with respect to the Ni–Fe-based alloy BM reference trend in the Larson–Miller representation, rather than by fixed temperature or stress thresholds. Due to confidentiality requirements, the specific temperature–stress combinations corresponding to the individual creep-rupture conditions are not disclosed. Accordingly, the three modes are used as descriptive regimes based on the degree of deviation in the welded-joint creep-rupture performance from the BM reference trend.

The observed variations are nevertheless associated with differences in microstructural evolution in the Ni–Fe-based alloy-side HAZ and BM. The following sections compare the fractographic and microstructural characteristics of the three regimes.

### 3.2. Fractographic Characteristics of Creep-Rupture Failure

**Figure 7:**
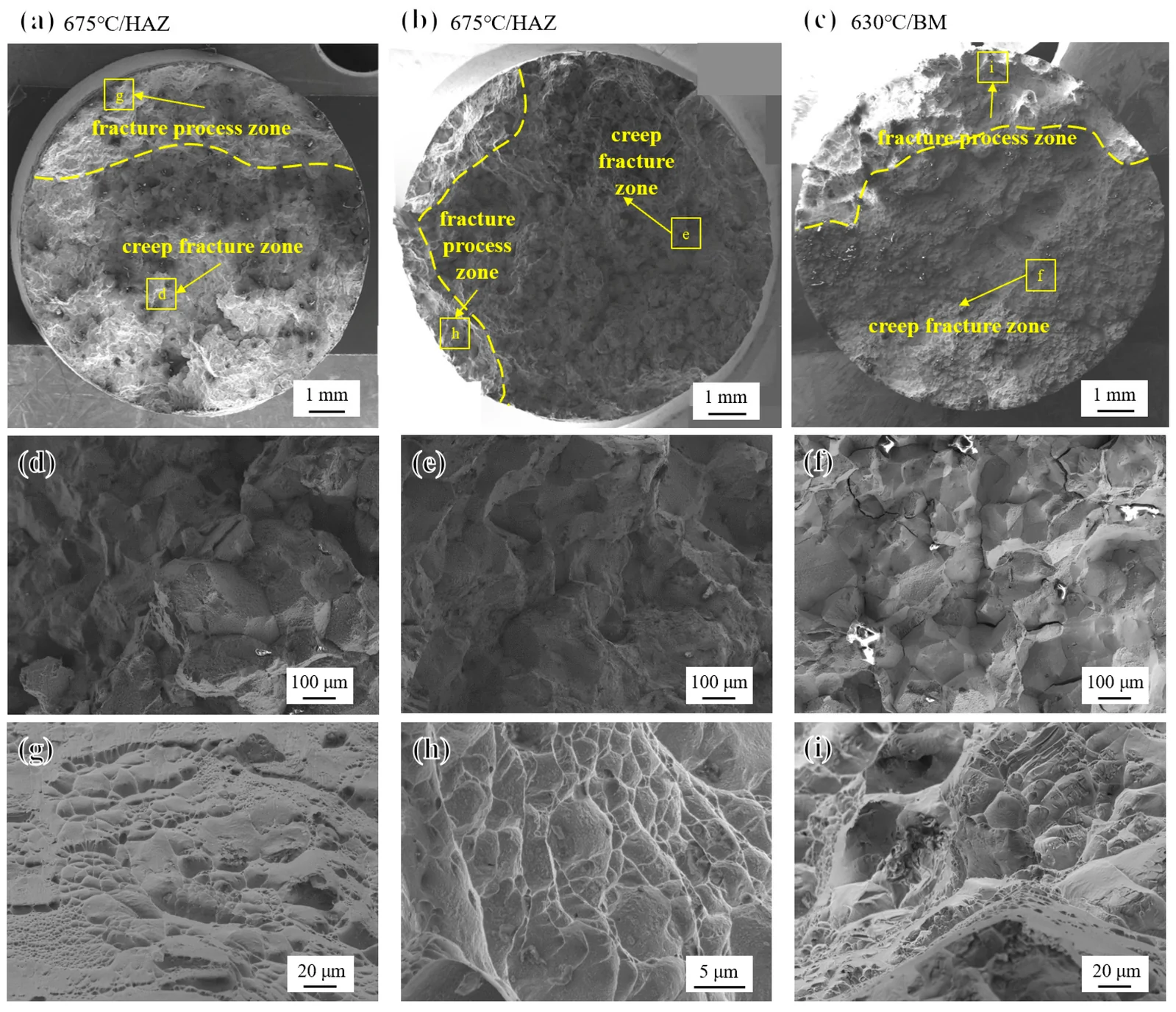
Fracture morphologies of the Ni–Fe-based alloy joints representative of the three failure regimes: (**a**) overall fracture morphology of a 675 °C/HAZ specimen; (**b**) overall fracture morphology of another 675 °C/HAZ specimen; (**c**) overall fracture morphology of a 630 °C/BM specimen; (**d**) enlarged view of the creep fracture zone marked in (**a**); (**e**) enlarged view of the creep fracture zone marked in (**b**); (**f**) enlarged view of the creep fracture zone marked in (**c**); (**g**) enlarged view of the fracture process zone marked in (**a**); (**h**) enlarged view of the fracture process zone marked in (**b**); (**i**) enlarged view of the fracture process zone marked in (**c**).

Figure 7 presents representative fracture morphologies of Ni–Fe-based alloy/Inconel 617 dissimilar welded joints tested under different stress conditions. All specimens exhibited pronounced intergranular fracture features and low fracture ductility, consistent with the measured elongation after rupture and reduction in area. Grain-boundary facets, secondary cracks, and creep cavities were widely observed on the fracture surfaces, indicating that cracks propagated predominantly along grain boundaries and ultimately caused joint failure.

As shown in Figure 7a–c, the fracture surfaces in all three modes, irrespective of whether failure occurred in the HAZ or BM, consisted primarily of a creep-fracture region and a final tearing region. The creep-fracture region formed through the accumulation and propagation of creep damage during high-temperature exposure, whereas the tearing region developed during the final fracture stage. Figure 7d–f show a predominantly faceted intergranular morphology in the creep-fracture region, demonstrating that creep-rupture failure occurred mainly along grain boundaries. Grain boundaries were therefore weaker than grain interiors at an elevated temperature and were susceptible to grain-boundary sliding, cavity formation, and cracking; the severity of these processes was closely related to grain-boundary precipitates. In contrast, the tearing regions in Figure 7g–i contained numerous dimples and exhibited typical ductile-fracture characteristics.

Although intergranular fracture was common to all three regimes, the distribution and severity of grain-boundary damage differed. The microstructures near the fracture locations were therefore examined to evaluate how DP and other local microstructural features were associated with the observed behavior.

### 3.3. Microstructural Characteristics and Failure Mechanisms of the Different Failure Modes

#### 3.3.1. Mode I: Comparable-Life Regime

**Figure 8:**
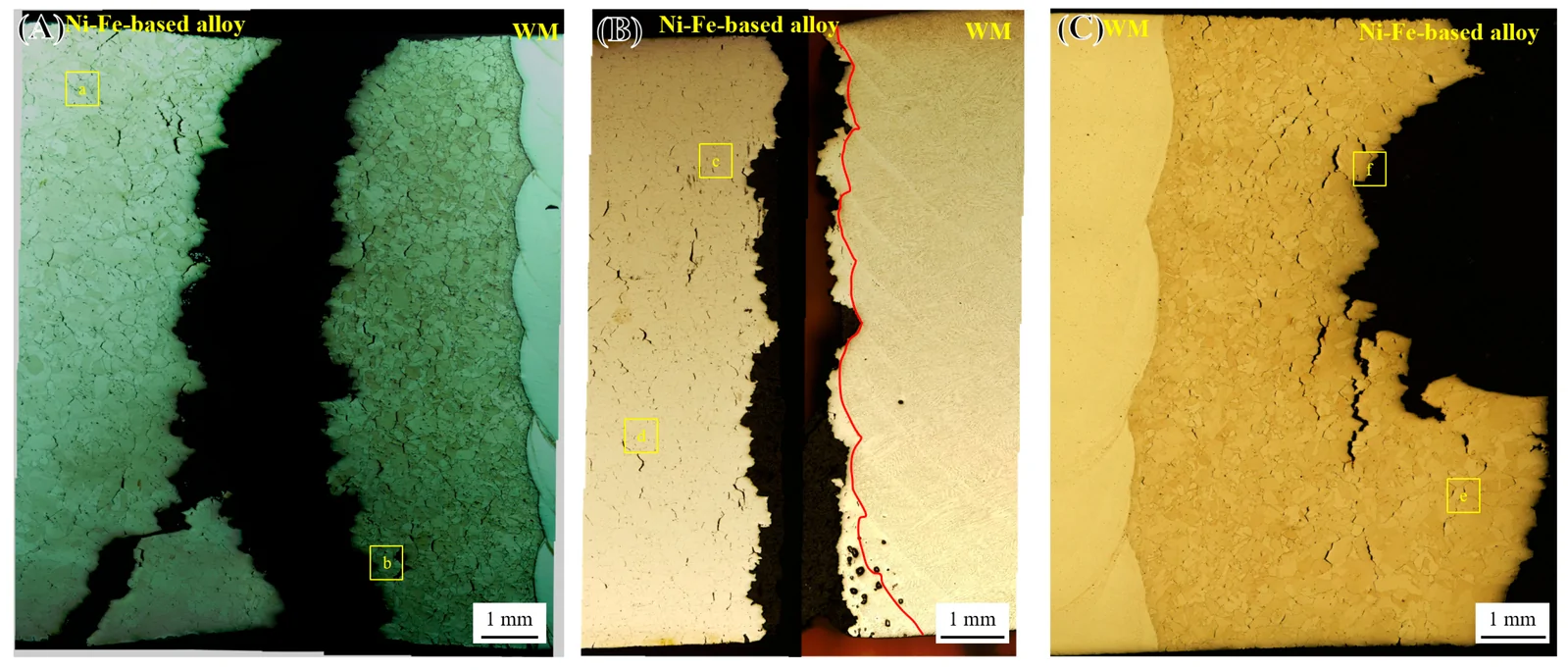
Metallographic cross-sections of creep-ruptured specimens representative of Mode I: (**A**) specimen tested at 675 °C with fracture occurring in the HAZ; (**B**) specimen tested at 700 °C with fracture occurring in the HAZ; (**C**) specimen tested at 700 °C with fracture occurring in the BM. The lowercase labels (a,b), (c,d), and (e,f) indicate representative regions selected from (**A**), (**B**), and (**C**), respectively, for further SEM examination shown in Figure 9. WM, weld metal; HAZ, heat-affected zone; BM, base metal.

Figure 8 shows metallographic cross-sections near the fracture locations of Mode I specimens. Intergranular creep cracks were present in both the HAZ and BM. The HAZ contained numerous intergranular cracks, while intergranular and secondary cracks were also observed in the BM adjacent to the fracture. These observations indicate substantial grain-boundary damage in both regions.

**Figure 9:**
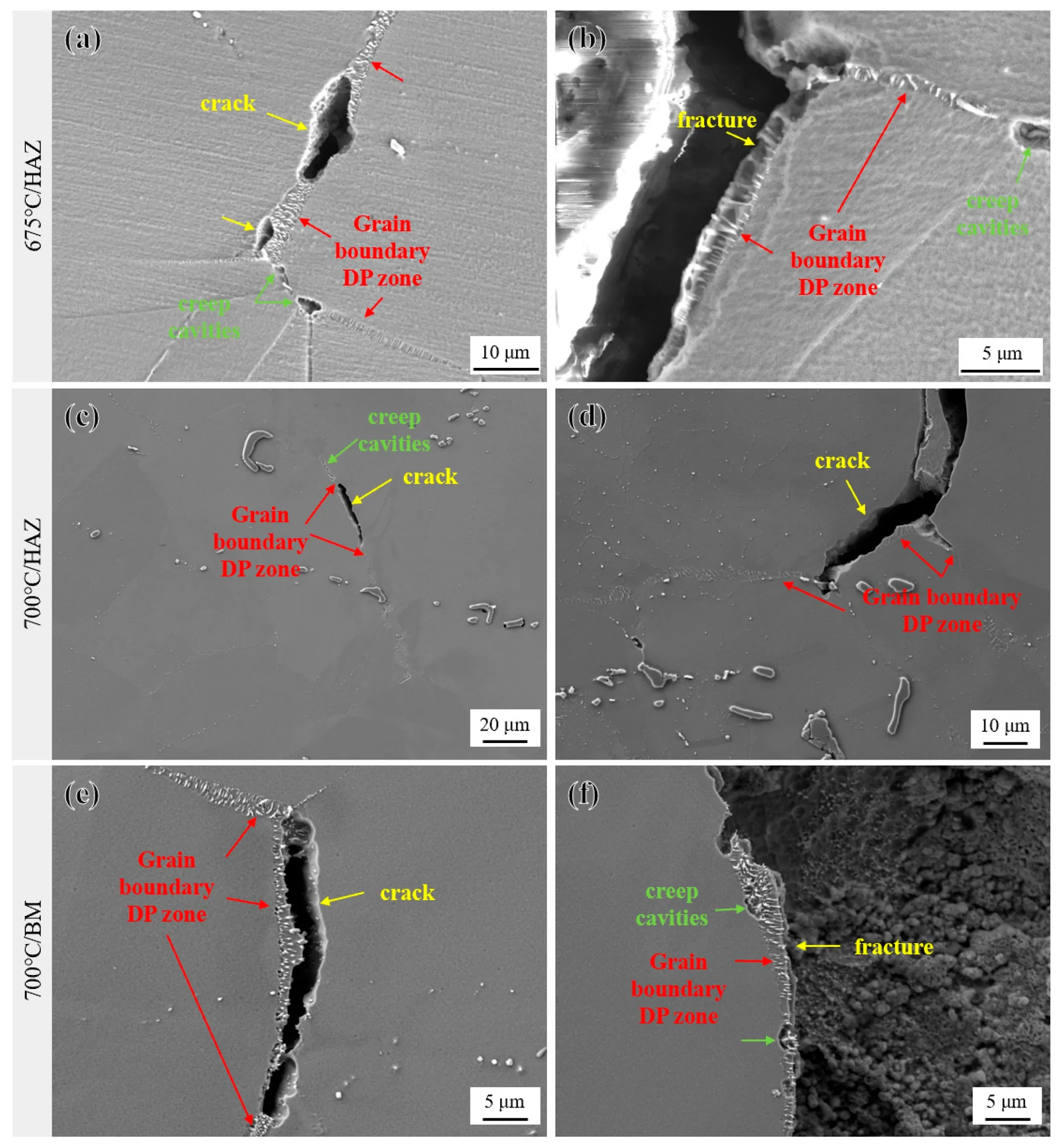
SEM micrographs of creep-ruptured Ni–Fe-based alloy joints representative of Mode I: (**a**) grain-boundary DP zone associated with a crack and creep cavities in the 675 °C/HAZ specimen; (**b**) grain-boundary DP zone adjacent to the fracture surface, accompanied by creep cavities, in the 675 °C/HAZ specimen; (**c**) grain-boundary DP zone associated with a crack and creep cavities in the 700 °C/HAZ specimen; (**d**) crack propagation along a grain-boundary DP zone in the 700 °C/HAZ specimen; (**e**) crack associated with a grain-boundary DP zone in the 700 °C/BM specimen; (**f**) grain-boundary DP zone adjacent to the fracture surface, accompanied by creep cavities, in the 700 °C/BM specimen. HAZ, heat-affected zone; BM, base metal; DP, discontinuous precipitation.

**Figure 10:**
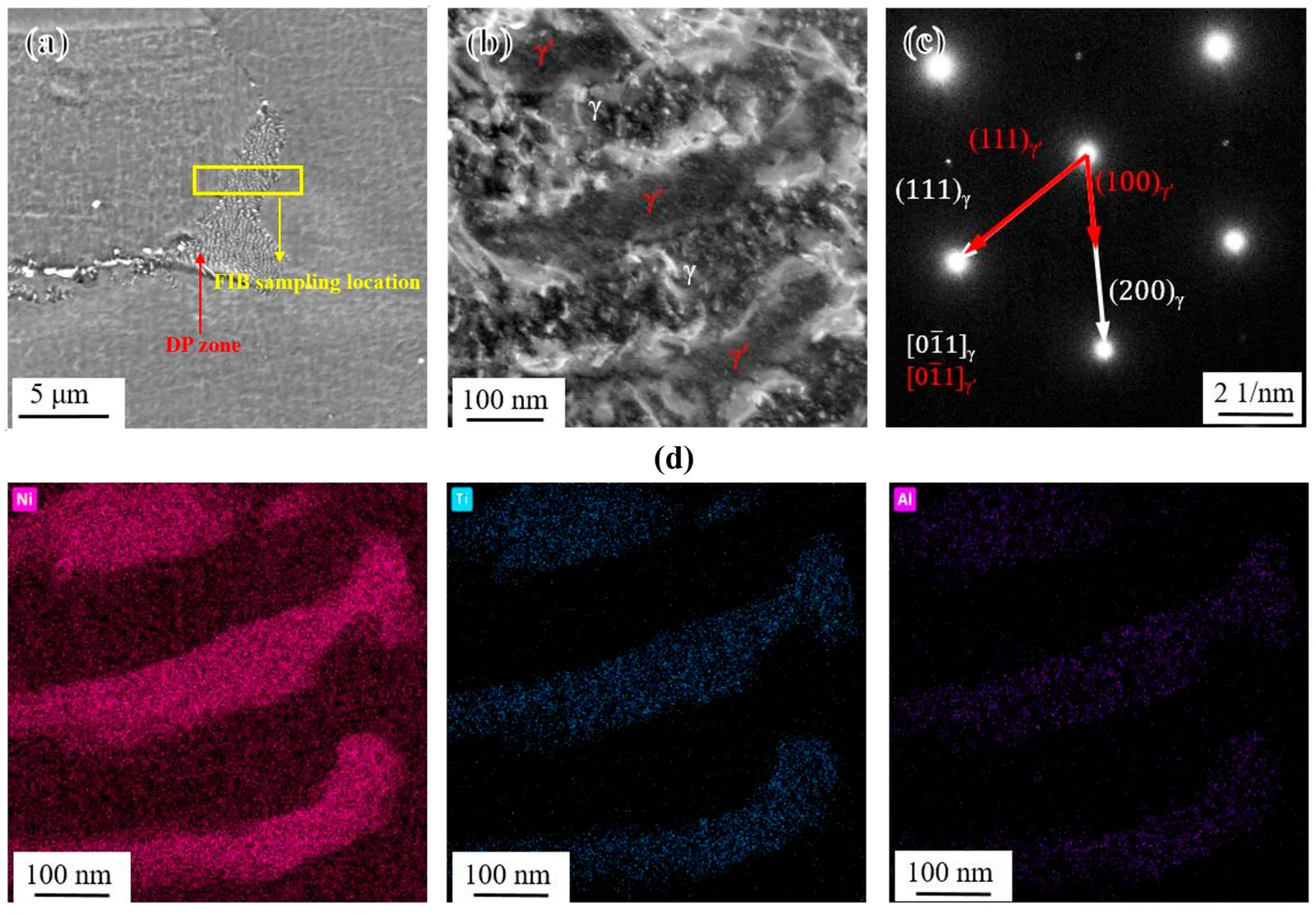
TEM characterization and phase identification of the rod-like precipitates in a representative grain-boundary DP region: (**a**) SEM image showing the DP region and the site-specific FIB sampling location; (**b**) bright-field TEM image showing the rod-like γ′ precipitates; (**c**) corresponding selected-area electron diffraction (SAED) pattern; and (**d**) EDS elemental maps of Ni, Ti, and Al.

To clarify the crack-formation mechanism, representative regions in Figure 8 were further examined by SEM, as shown in Figure 9. Pronounced grain-boundary DP was observed adjacent to the creep-rupture surfaces, intergranular cracks, and creep cavities. The DP regions consisted mainly of coarsened rod-like γ′ precipitates and PFZs and were widely associated with creep cavities and microcracks. To further identify rod-like precipitates within the grain-boundary DP regions, a representative DP region was characterized by site-specific TEM, as shown in Figure 10. The sampling location is indicated in Figure 10a. The bright-field TEM image (Figure 10b) reveals rod-like precipitates within the DP region. The corresponding SAED pattern (Figure 10c) shows that the rod-like precipitates exhibit an ordered FCC-related structure and maintain a coherent crystallographic relationship with the γ matrix. EDS elemental mapping (Figure 10d) further demonstrates enrichment of Ni, Ti, and Al in the rod-like precipitates. Based on the combined crystallographic and compositional evidence, these rod-like precipitates were identified as coarsened γ′ precipitates. No evidence of η-Ni_3_Ti was detected in the examined DP region. These observations indicate that grain boundaries affected by DP possess lower microstructural stability than unaffected boundaries and are more susceptible to grain-boundary sliding and creep-damage accumulation during creep exposure. Creep cavities preferentially nucleated in DP regions and subsequently grew and coalesced into intergranular cracks, ultimately producing intergranular fracture.

Although the Ni–Fe-based alloy base metal did not exhibit obvious grain-boundary DP before creep testing, severe DP progressively developed in the BM during long-term exposure at 675–700 °C. At 700 °C, the high temperature promoted rapid DP formation; at 675 °C, substantial DP developed despite the lower temperature because of the longer exposure time, which likewise allowed DP to develop extensively. Consequently, under the low-stress conditions corresponding to Mode I, the HAZ and BM ultimately exhibited comparable degrees of grain-boundary DP and similar grain-boundary damage. Because their grain-boundary stabilities were similar, their creep-rupture resistances were also comparable, and the joint and Ni–Fe-based alloy base metal exhibited similar rupture lives. Both the HAZ and BM could therefore act as creep-critical regions, giving rise to the random failure locations characteristic of Mode I in Figure 6.

#### 3.3.2. Mode II: Moderately Reduced-Life Regime

**Figure 11:**
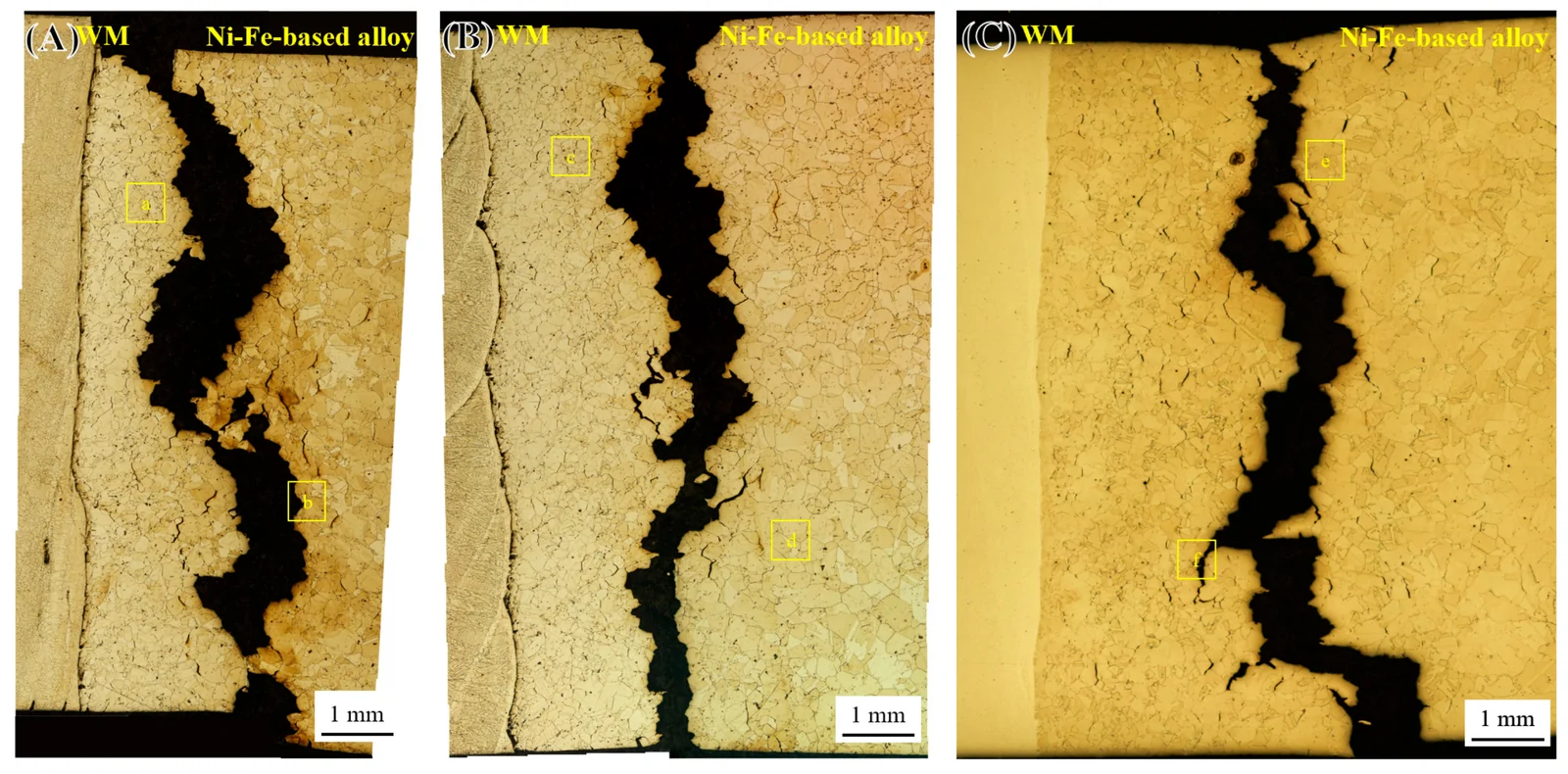
Metallographic cross-sections of creep-ruptured specimens representative of Mode II: (**A**) specimen tested at 650 °C with fracture occurring in the HAZ; (**B**) another specimen tested at 650 °C with fracture occurring in the HAZ; (**C**) specimen tested at 675 °C with fracture occurring in the HAZ. The lowercase labels (a,b), (c,d), and (e,f) indicate representative regions selected from (**A**), (**B**), and (**C**), respectively, for further SEM examination shown in Figure 12. WM, weld metal; HAZ, heat-affected zone.

Figure 11 shows representative microstructures near the fracture surfaces of Mode II Ni–Fe-based alloy/Inconel 617 dissimilar welded joints. All three specimens fractured in the fine-grained region of the Ni–Fe-based alloy-side HAZ. Numerous intergranular cracks were concentrated in the HAZ adjacent to the fracture, whereas almost no obvious creep cracks were observed in the BM. Under intermediate applied stresses, creep damage was therefore strongly localized in the HAZ.

**Figure 12:**
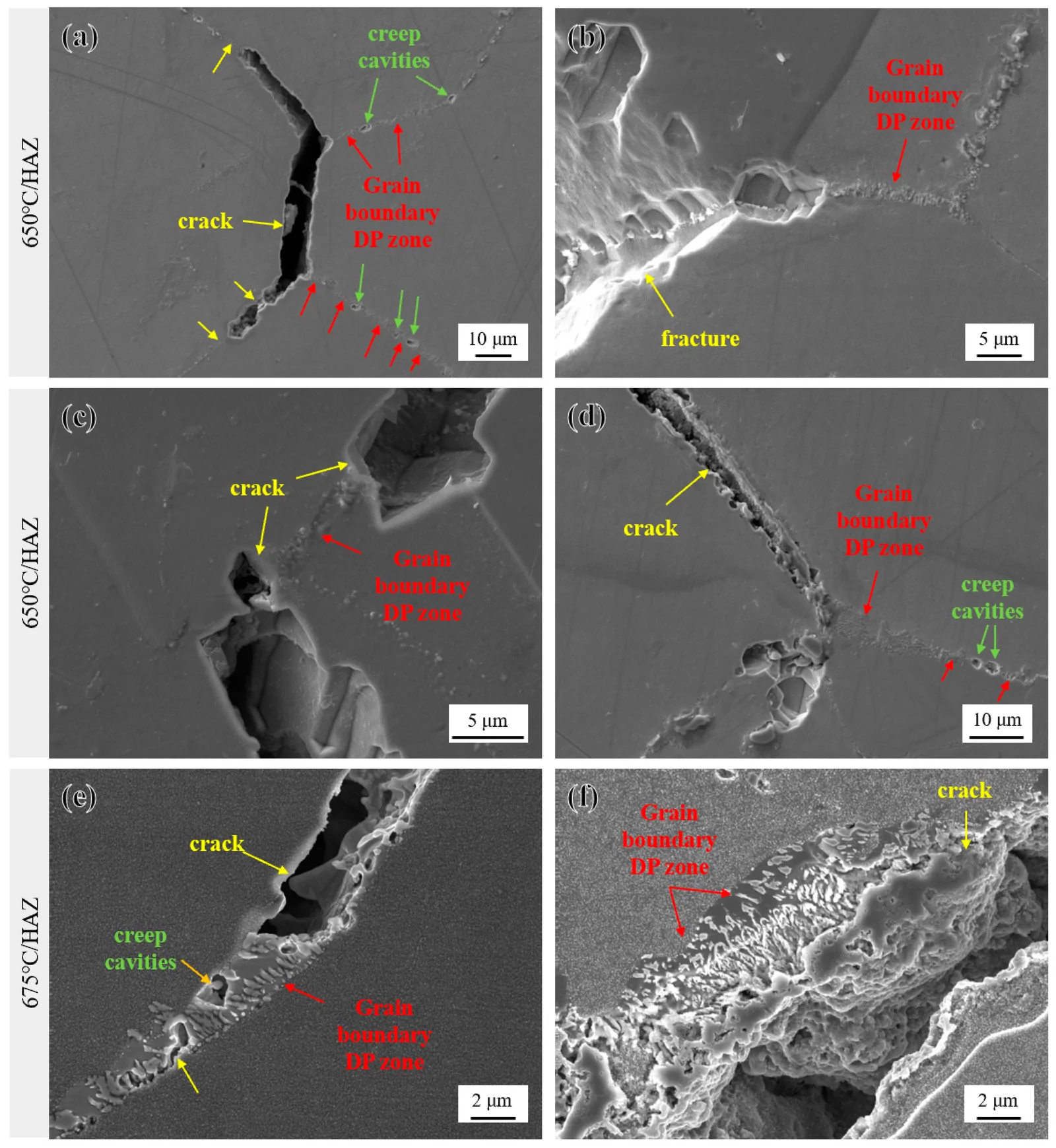
SEM micrographs of creep-ruptured Ni–Fe-based alloy joints representative of Mode II: (**a**) grain-boundary DP zone associated with a crack and creep cavities in a 650 °C/HAZ specimen; (**b**) grain-boundary DP zone adjacent to the fracture surface in the same 650 °C/HAZ specimen; (**c**) grain-boundary DP zone associated with cracks in another 650 °C/HAZ specimen; (**d**) crack propagation along a grain-boundary DP zone accompanied by creep cavities in the same 650 °C/HAZ specimen; (**e**) crack and creep cavities associated with a grain-boundary DP zone in the 675 °C/HAZ specimen; (**f**) grain-boundary DP zone adjacent to the fracture surface in the 675 °C/HAZ specimen. HAZ, heat-affected zone; DP, discontinuous precipitation.

Representative regions in Figure 11 were further examined by SEM to clarify the failure mechanism, as shown in Figure 12. As in Mode I, extensive grain-boundary DP was observed adjacent to the creep-rupture surfaces, intergranular cracks, and creep cavities. The cavities were mainly located within DP regions or at interfaces between DP regions and grain-boundary carbides, confirming that DP regions were preferential sites for creep-damage initiation and accumulation. The fundamental failure mechanism under intermediate stress was therefore similar to that in Mode I: grain-boundary DP weakened the boundaries and promoted cavity nucleation, growth, and coalescence into cracks.

Unlike Mode I, however, all three Mode II specimens tested at intermediate stress failed in the HAZ, and their creep-rupture lives were shorter than that of the Ni–Fe-based alloy base metal. This behavior is attributed to the relatively lower test temperatures or shorter rupture times under these conditions. Consequently, only limited grain-boundary DP developed in the BM during creep, whereas extensive DP had already formed in the HAZ during the welding thermal cycle and post-weld heat treatment. By the later stages of creep, the extent of grain-boundary DP in the HAZ was therefore greater than that in the BM, resulting in lower grain-boundary stability and preferential accumulation of creep damage in the HAZ.

In addition, the fine-grained HAZ has a higher grain-boundary density and is therefore more susceptible to grain-boundary sliding and strain localization under combined high-temperature and stress conditions, which further promotes cavity formation and crack propagation. The combined effects of grain-boundary DP and the fine-grained microstructure make the HAZ the weakest region of the joint. Accordingly, in Mode II, the creep-rupture life of the joint was slightly shorter than that of the Ni–Fe-based alloy base metal, corresponding to the moderately degraded behavior shown in Figure 6.

#### 3.3.3. Mode III: Markedly Reduced-Life Regime

**Figure 13:**
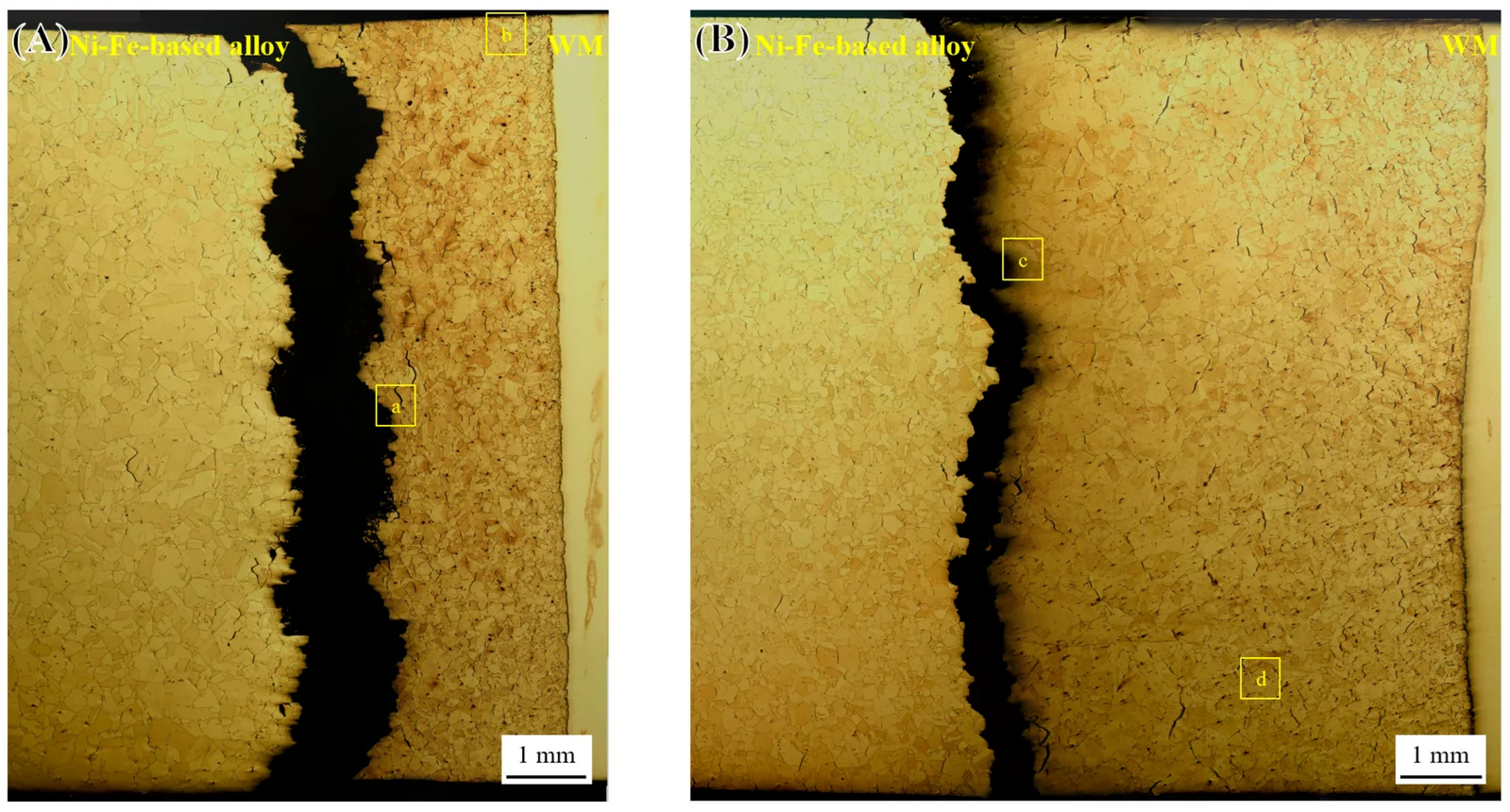
Metallographic cross-sections of creep-ruptured specimens representative of Mode III: (**A**) specimen tested at 630 °C with fracture occurring in the HAZ; (**B**) specimen tested at 630 °C with fracture occurring in the BM. The lowercase labels (a,b) and (c,d) indicate representative regions selected from (**A**) and (**B**), respectively, for further SEM examination shown in Figure 14. WM, weld metal; HAZ, heat-affected zone; BM, base metal.

Figure 13 shows representative microstructures near the fracture surfaces of Mode III Ni–Fe-based alloy/Inconel 617 dissimilar welded joints. The specimen tested at 630 °C fractured in the fine-grained Ni–Fe-based alloy-side HAZ and contained numerous intergranular secondary cracks around the fracture. Although the specimen tested at 630 °C ultimately failed in the BM, numerous intergranular cracks were also present in its HAZ. Overall, Mode III failure remained concentrated on the Ni–Fe-based alloy side, particularly in the fine-grained HAZ.

**Figure 14:**
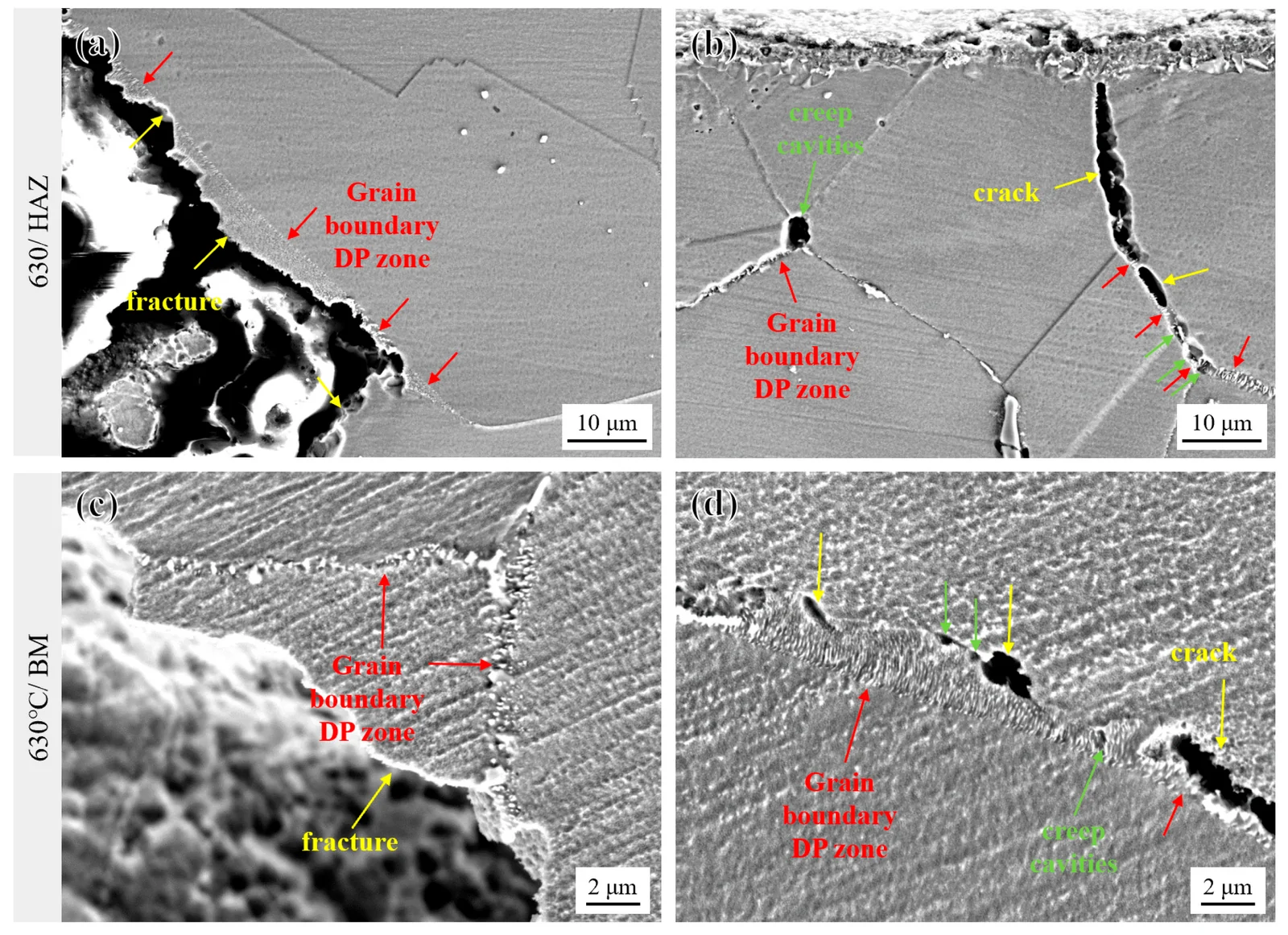
SEM micrographs of creep-ruptured Ni–Fe-based alloy joints representative of Mode III: (**a**) grain-boundary DP zone adjacent to the fracture surface in the 630 °C/HAZ specimen; (**b**) grain-boundary DP zone associated with a crack and creep cavities in the 630 °C/HAZ specimen; (**c**) grain-boundary DP zone adjacent to the fracture surface in the 630 °C/BM specimen; (**d**) grain-boundary DP zone associated with a crack and creep cavities in the 630 °C/BM specimen. HAZ, heat-affected zone; BM, base metal; DP, discontinuous precipitation.

Four representative regions marked by yellow boxes in the metallographic cross-sections in Figure 13 were further examined by SEM, as shown in Figure 14. Similar to Modes I and II, extensive grain-boundary DP was observed adjacent to the creep-rupture surfaces, intergranular cracks, and creep cavities. The creep cavities preferentially nucleated within DP regions or at interfaces between DP regions and grain-boundary carbides and then grew and coalesced into intergranular cracks. Grain-boundary DP regions were therefore the principal sites of preferential creep-damage accumulation and crack propagation.

Compared with the first two failure modes, Mode III corresponded to the lowest test temperatures and shortest rupture times. Although DP also developed in the BM, its extent was markedly lower than that in the HAZ. The difference in grain-boundary stability between the HAZ and BM therefore increased further, making the HAZ the weakest region of the joint. Extensive, nearly continuous DP regions and PFZs substantially reduced the load-bearing capacity of the grain boundaries, causing rapid cavity nucleation, growth, and coalescence into cracks. Consequently, the creep-rupture life of the joint was substantially shorter than that of the Ni–Fe-based alloy base metal, corresponding to the severely degraded behavior shown in Figure 6.

It is noteworthy that the specimen tested at 630 °C ultimately failed in the BM rather than the HAZ. Fractographic and metallographic observations showed that the fracture was located in a mixed-grain region of the BM with pronounced local variations in grain size. Such grain-size heterogeneity may impair local strain compatibility and promote stress concentration, thereby reducing local creep-rupture resistance. A pronounced grain-size dependence of creep-rupture behavior has also been reported for precipitation-strengthened Ni-based superalloys [25]. Failure of this specimen in the BM was therefore primarily associated with the mixed-grain microstructure rather than being directly governed by grain-boundary DP. Nevertheless, extensive intergranular cracking and grain-boundary DP were still observed in its HAZ, indicating that in the absence of local defects such as mixed grains, high-stress failure would remain controlled primarily by grain-boundary DP damage in the HAZ.

#### 3.3.4. Effect of Grain-Boundary DP Evolution on Failure-Mode Transition

The preceding results suggest that the different creep-rupture failure regimes of Ni–Fe-based alloy/Inconel 617 dissimilar welded joints are closely associated with differences in the extent of grain-boundary DP evolution between the HAZ and BM. Unlike the Ni–Fe-based alloy base metal, the HAZ had already developed grain-boundary DP during standard post-weld heat treatment and therefore possessed lower initial grain-boundary stability [26,27]. During subsequent creep exposure, DP continued to evolve in both the HAZ and BM. The relative extent of this evolution depended on temperature, stress, and exposure time, giving rise to the different failure modes.

In Mode I, the relatively high test temperature or long exposure time allowed severe grain-boundary DP to develop in the BM during the later stages of creep, so that its DP extent approached that of the HAZ. The two regions consequently exhibited similar grain-boundary stability and creep-damage levels, and either region could become the final failure location. The joint therefore exhibited a creep-rupture life comparable to that of the Ni–Fe-based alloy base metal.

In Mode II, the lower test temperature or shorter exposure time limited the development of grain-boundary DP in the BM relative to the HAZ, producing a clear mismatch in grain-boundary stability [28]. Creep cavities preferentially nucleated and accumulated in HAZ DP regions, causing the HAZ to reach the critical damage state first. The creep-rupture life of the joint was therefore slightly shorter than that of the Ni–Fe-based alloy base metal, and failure occurred primarily in the fine-grained HAZ.

In Mode III, the still lower test temperature and substantially shorter exposure time strongly suppressed DP development in the BM, whereas the HAZ retained extensive pre-existing DP. The grain-boundary stability mismatch between the HAZ and BM was therefore greatest, making the HAZ the most vulnerable region. Rapid nucleation, growth, and coalescence of numerous creep cavities produced cracks and markedly shortened the creep-rupture life relative to the Ni–Fe-based alloy base metal. In the single specimen that failed in the BM, fracture was controlled by local microstructural defects such as mixed grains rather than by the characteristic DP-dominated mechanism.

Grain-boundary DP evolution is therefore closely associated with the difference in grain-boundary stability between the HAZ and BM [28]. This difference, in turn, contributes to the localization of creep damage and is closely linked to the transition among the creep-rupture failure regimes of Ni–Fe-based alloy/Inconel 617 dissimilar welded joints. As the difference in DP extent between the HAZ and BM evolves from negligible to pronounced, the failure behavior changes sequentially from Mode I (Comparable-Life Regime) to Mode II (Moderately Reduced-Life Regime) and then to Mode III (Markedly Reduced-Life Regime).

**Figure 15:**
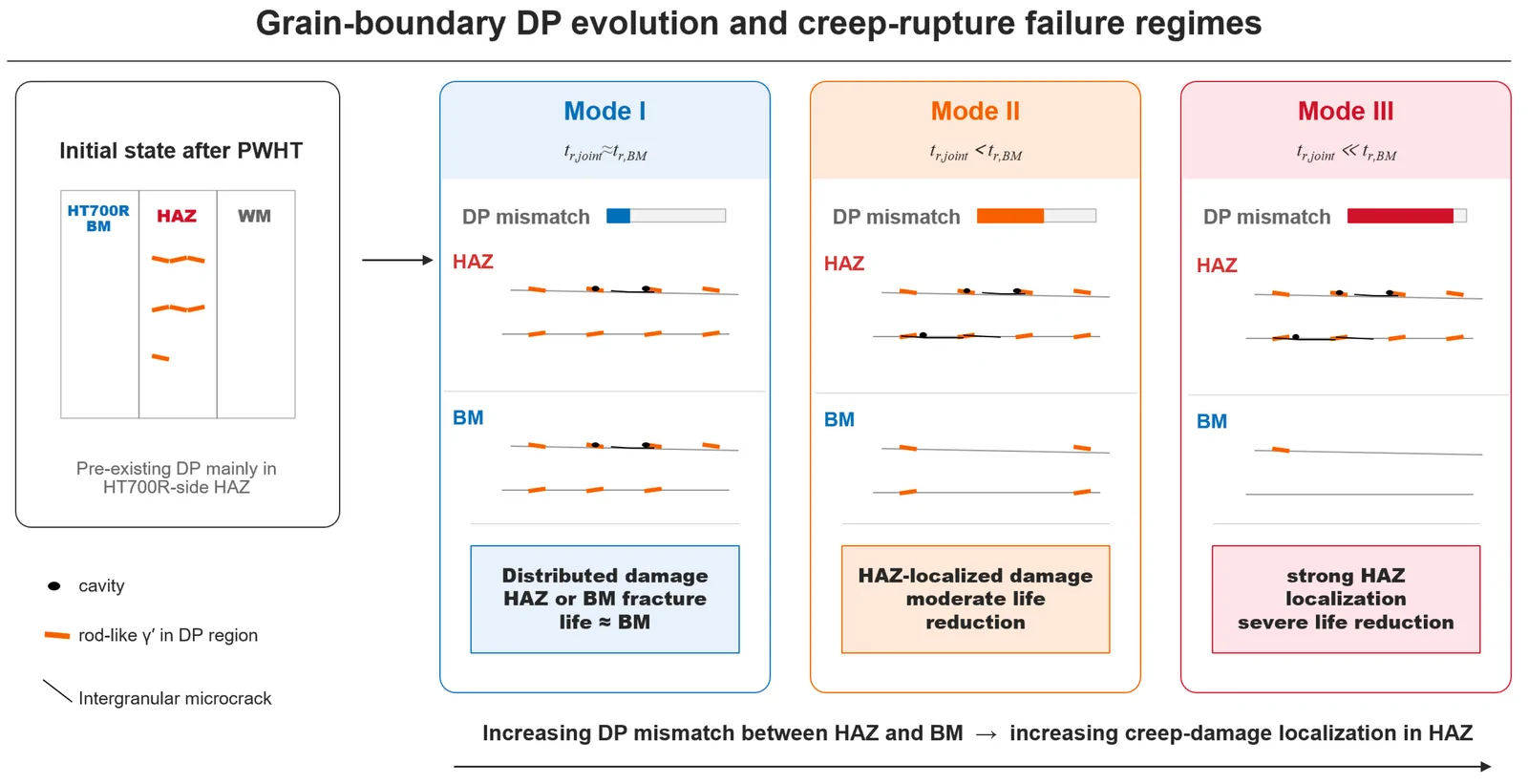
Schematic illustration of the relationship between grain-boundary DP evolution and the transition of creep-rupture failure modes in Ni–Fe-based alloy/Inconel 617 dissimilar welded joints.

A conceptual framework summarizing this interpretation is shown in Figure 15. DP preferentially developed in the Ni–Fe-based alloy-side HAZ during welding and post-weld heat treatment. During creep exposure, DP continued to evolve in both the HAZ and BM. Temperature, applied stress, and exposure time altered the relative extent of DP in the two regions and, consequently, the degree of creep-damage localization. Grain-boundary DP therefore provides an important microstructural link between thermal–mechanical exposure and the observed creep-rupture behavior of the joint.

### 3.4. Formation Conditions and Evolution of Grain-Boundary DP in Ni–Fe-Based Alloy

The preceding results show that grain-boundary DP is an important microstructural degradation process controlling the creep-rupture performance of the Ni–Fe-based alloy because its formation and development directly affect grain-boundary stability and creep-damage accumulation. The formation conditions and evolution of grain-boundary DP in the Ni–Fe-based alloy were therefore investigated further. Quantitative statistical analyses were used to systematically evaluate the effects of temperature, stress, and time and to provide a basis for clarifying the formation mechanism [29].

#### 3.4.1. Finite Element Simulation Results

**Figure 16:**
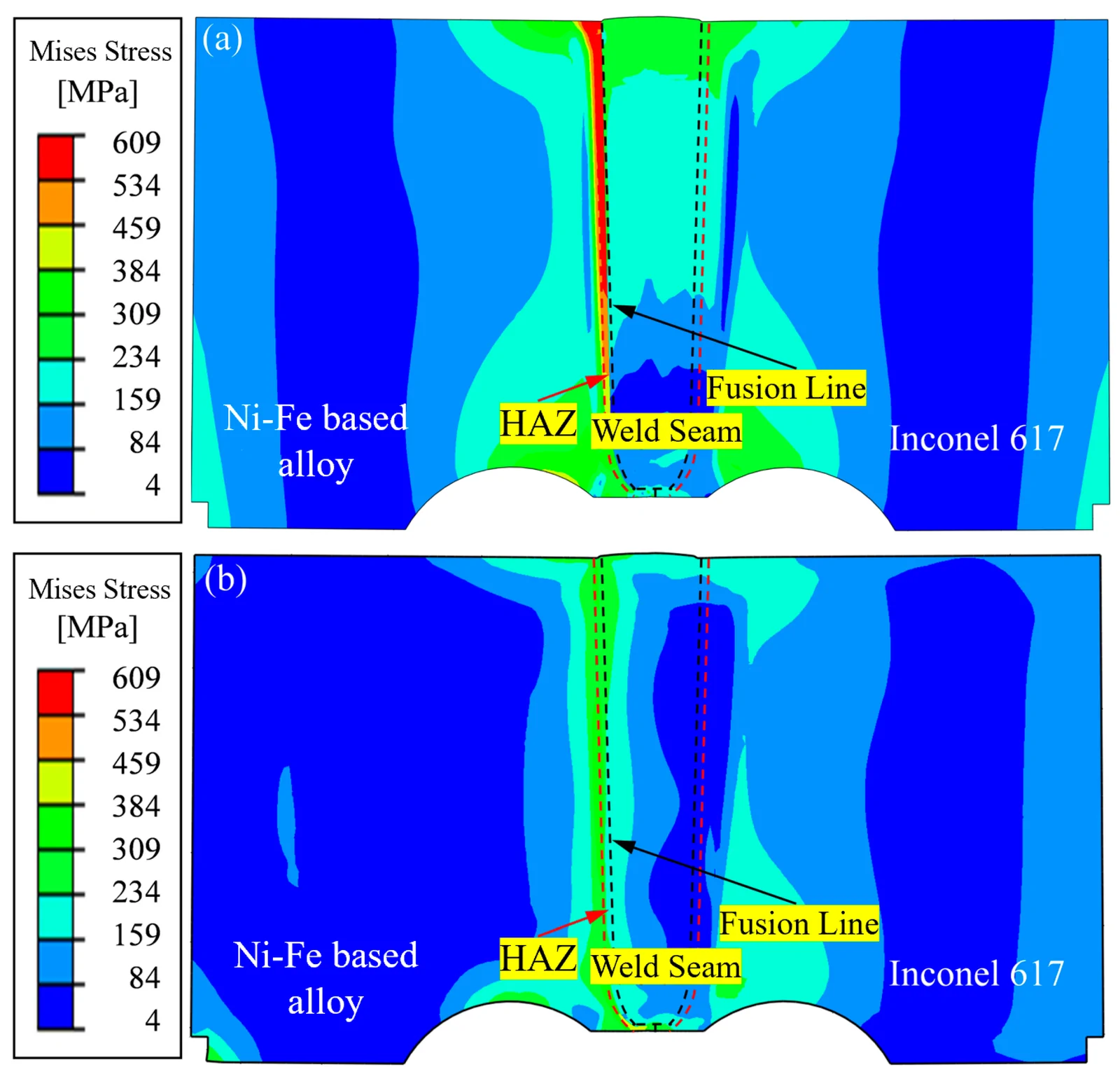
Von Mises stress distributions in the Ni–Fe-based alloy/Inconel 617 dissimilar superalloy ring-welded joint: (**a**) as-welded condition; (**b**) after solution treatment.

Figure 16 shows the predicted Von Mises residual-stress distributions in the Ni–Fe-based alloy/Inconel 617 ring-shaped dissimilar superalloy welded joint after solution treatment. Comparison with the as-welded condition in Figure 16a shows that solution treatment at 980 °C for 1 h substantially reduced the residual stress: the maximum Von Mises stress decreased from 609 MPa in the as-welded state to 396 MPa after solution treatment. Thus, solution treatment effectively relieved welding-induced residual stress, although a considerable residual-stress field remained in the joint.

**Figure 17:**
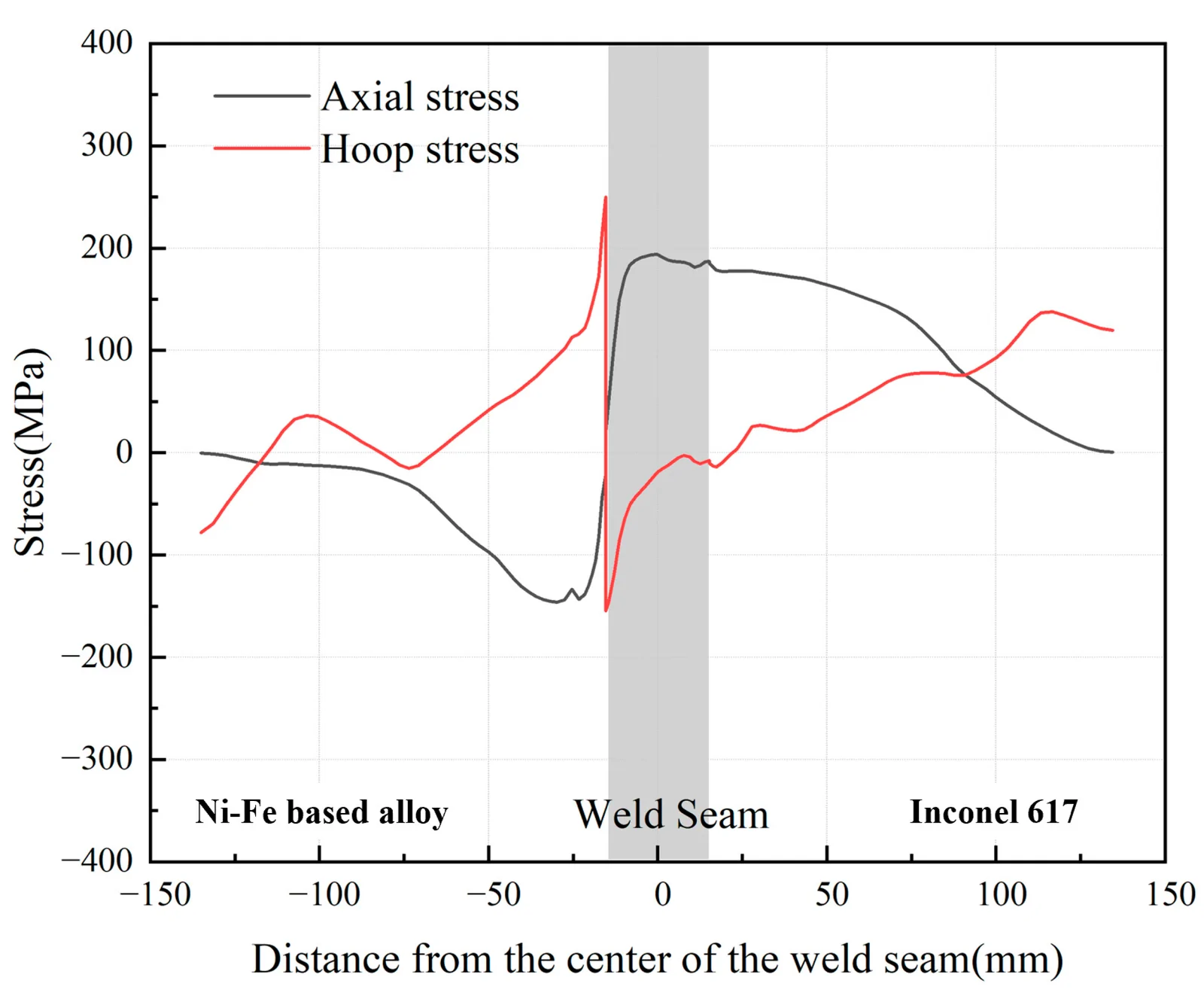
Numerically predicted residual stresses along an axial path on the outer surface of the ring-welded joint after solution treatment.

Figure 17 shows the predicted axial and hoop stresses along the selected surface path after solution treatment. The maximum axial compressive stress on the Ni–Fe-based alloy side was 162 MPa, and the axial distribution alternated between compressive and tensile regions. A steep hoop-stress gradient occurred near the Ni–Fe-based alloy-side fusion line. This nonuniform distribution reflects the combined effects of material–property mismatch and geometric constraint during cooling.

The finite element results show that a representative residual stress of approximately 150 MPa remained in the Ni–Fe-based alloy-side HAZ after solution treatment. Local stress can influence grain-boundary migration and elemental diffusion and can therefore affect the formation and evolution of grain-boundary DP. Accordingly, 150 MPa was selected as the representative stress for the interrupted creep tests to simulate the actual stress state of the welded-joint HAZ and to investigate the effects of temperature, stress, and time on grain-boundary DP evolution in the Ni–Fe-based alloy.

#### 3.4.2. Critical Conditions for Grain-Boundary DP Formation

**Figure 18:**
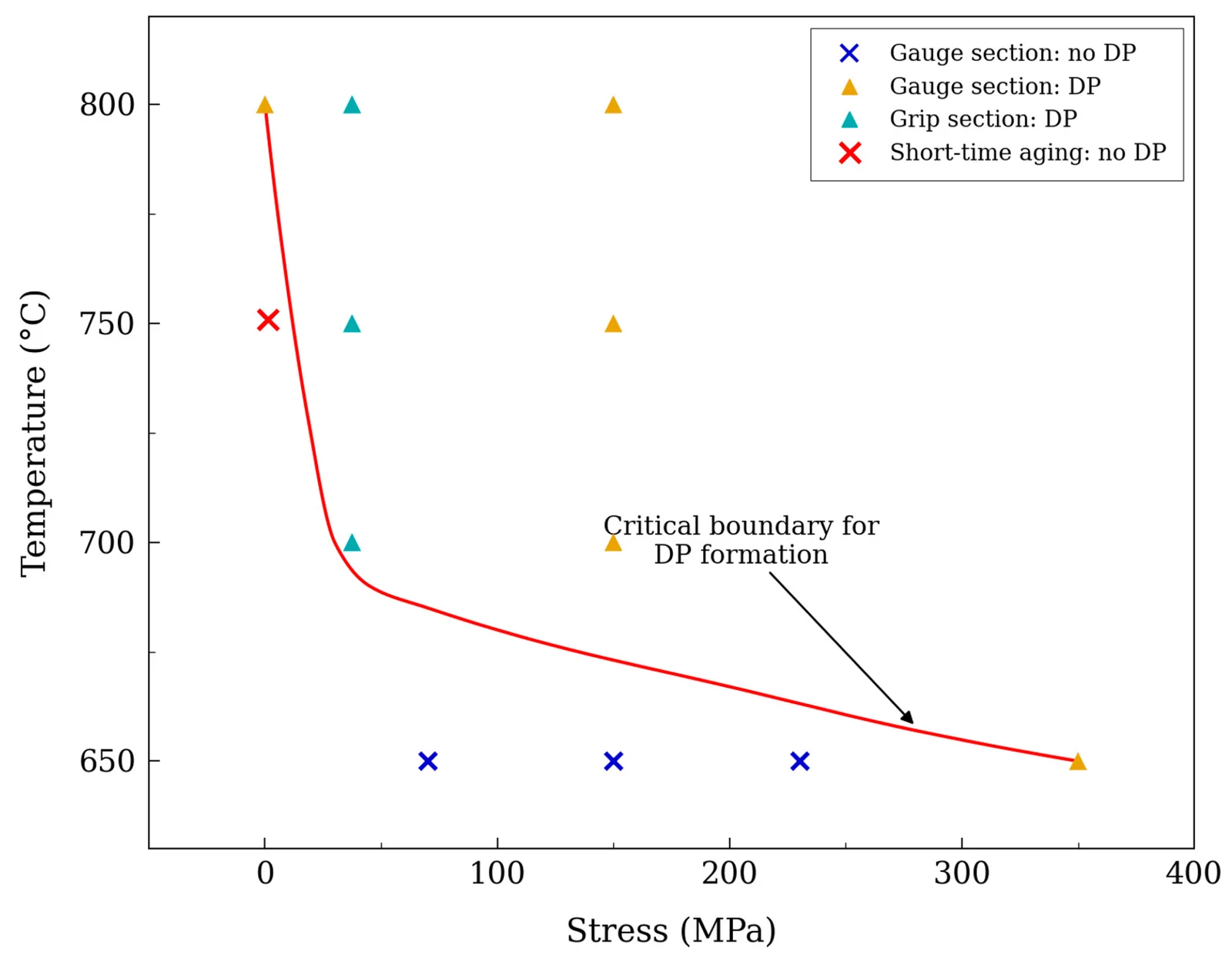
Critical conditions for grain-boundary DP formation in Ni–Fe-based alloy at different temperatures and stresses.

**Figure 19:**
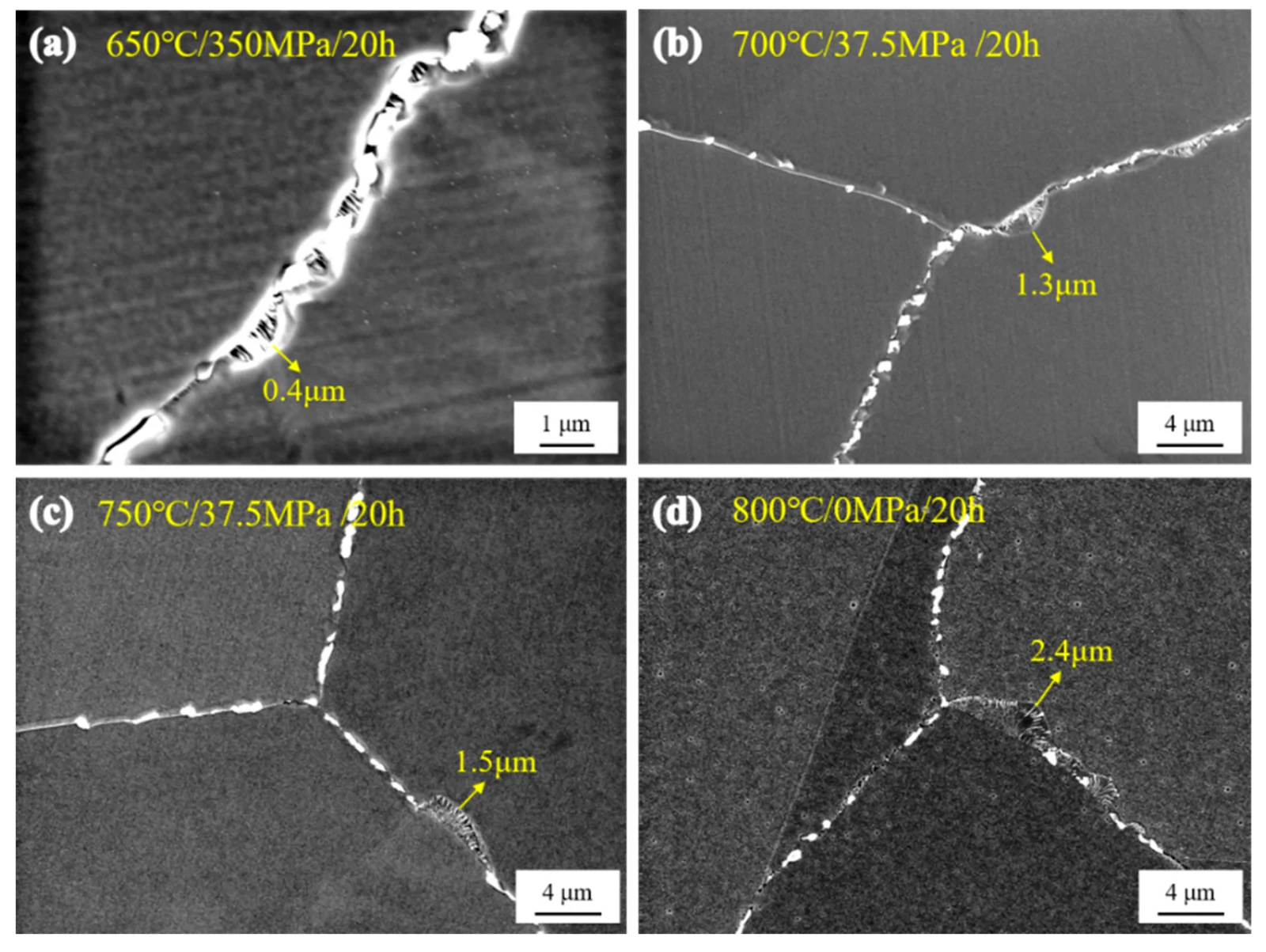
Representative grain-boundary DP morphologies under different temperature and stress conditions.

To determine the conditions required for grain-boundary DP formation in the Ni–Fe-based alloy, creep specimens exposed for 20 h at 650–800 °C under different stresses were examined, and a critical-condition map was constructed, as shown in Figure 18. The critical stress decreased markedly with increasing temperature, demonstrating that grain-boundary DP formation was jointly controlled by temperature and stress, with temperature playing the dominant role. At 650 °C, only the specimen tested at 350 MPa contained a few isolated DP regions with widths below 0.5 μm (Figure 19a); the critical stress for DP formation at this temperature was therefore approximately 350 MPa. At 700 and 750 °C, distinct grain-boundary DP was observed in both the 150 MPa gauge sections and the grip sections (Figure 19b,c). Combined with previous zero-stress isothermal-aging results at 700 and 750 °C, these observations indicate critical stresses below 37.5 MPa. At 800 °C, pronounced grain-boundary DP was observed not only in the grip section of the 150 MPa specimen but also in the zero-stress control specimen (Figure 19d), showing that DP formed without externally applied stress and that the critical stress decreased to 0 MPa.

These results demonstrate that grain-boundary DP in the Ni–Fe-based alloy is highly sensitive to temperature. As temperature increased, the promoting effect of applied stress became progressively weaker. At 800 °C, thermal activation alone provided a sufficient driving force for DP formation in the absence of external stress.

On this basis, the grain-boundary DP fraction, mean DP-region length, and mean DP-region width were used to quantitatively characterize DP evolution and to systematically evaluate the effects of temperature, stress, and time.

#### 3.4.3. Effect of Temperature on Grain-Boundary DP Evolution

**Figure 20:**
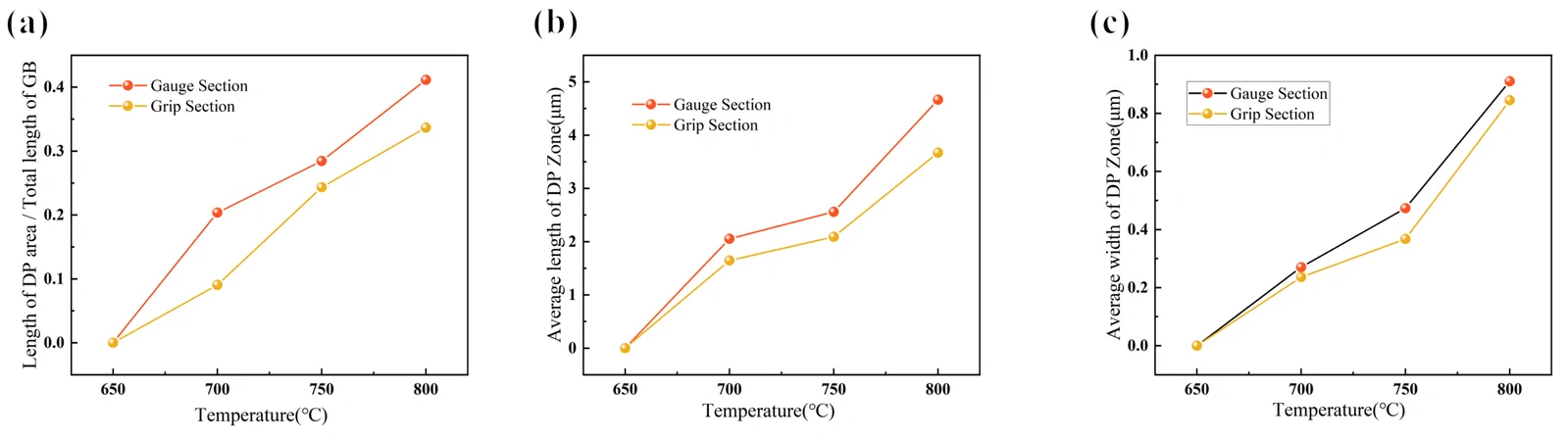
Temperature dependence of grain-boundary DP characteristics in the gauge and grip sections: (**a**) grain-boundary DP fraction; (**b**) mean DP-region length; (**c**) mean DP-region width.

To investigate the effect of temperature on grain-boundary DP in the Ni–Fe-based alloy, the grain-boundary DP fraction, mean DP-region length, and mean DP-region width were statistically evaluated in the gauge and grip sections of specimens exposed for 20 h at 650, 700, 750, and 800 °C under a gauge stress of 150 MPa. The results are shown in Figure 20.

As shown in Figure 20, the grain-boundary DP fraction, mean DP-region length, and mean DP-region width in both the gauge and grip sections increased markedly with temperature, and the two regions exhibited similar trends. The DP-region length and width were more temperature-sensitive than the DP fraction and increased more rapidly. Increasing temperature therefore promoted not only the formation of grain-boundary DP but also the expansion and coarsening of existing DP regions.

**Figure 21:**
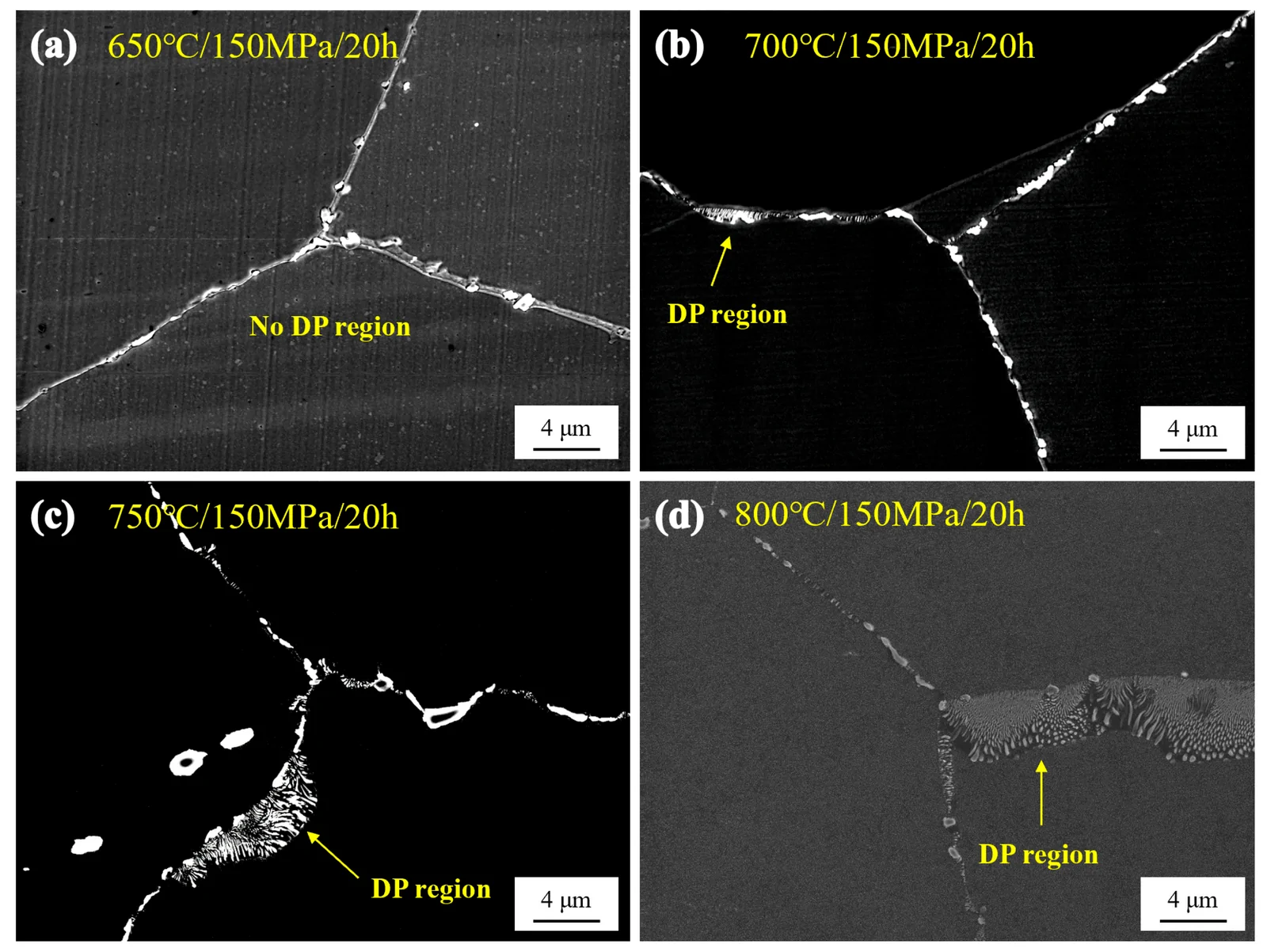
Representative grain-boundary DP regions in the gauge sections of Ni–Fe-based alloy specimens exposed for 20 h at 150 MPa and different temperatures.

Representative grain-boundary DP morphologies in the gauge sections at different temperatures are shown in Figure 21. As the temperature increased from 650 to 800 °C, the DP regions enlarged substantially and evolved from isolated local regions into large, nearly continuous coarsened regions. The higher temperature enhanced elemental diffusion and grain-boundary migration and thereby accelerated the formation and growth of grain-boundary DP.

Together with the critical-condition analysis in Section 3.4.2, these results show that temperature determines not only whether grain-boundary DP can form but also its evolution rate and final extent. As temperature increased, the critical stress for DP formation decreased markedly and the DP regions enlarged rapidly. Temperature is therefore the dominant factor controlling grain-boundary DP in the Ni–Fe-based alloy, whereas applied stress plays a secondary promoting role.

#### 3.4.4. Effect of Stress on Grain-Boundary DP Evolution

**Figure 22:**
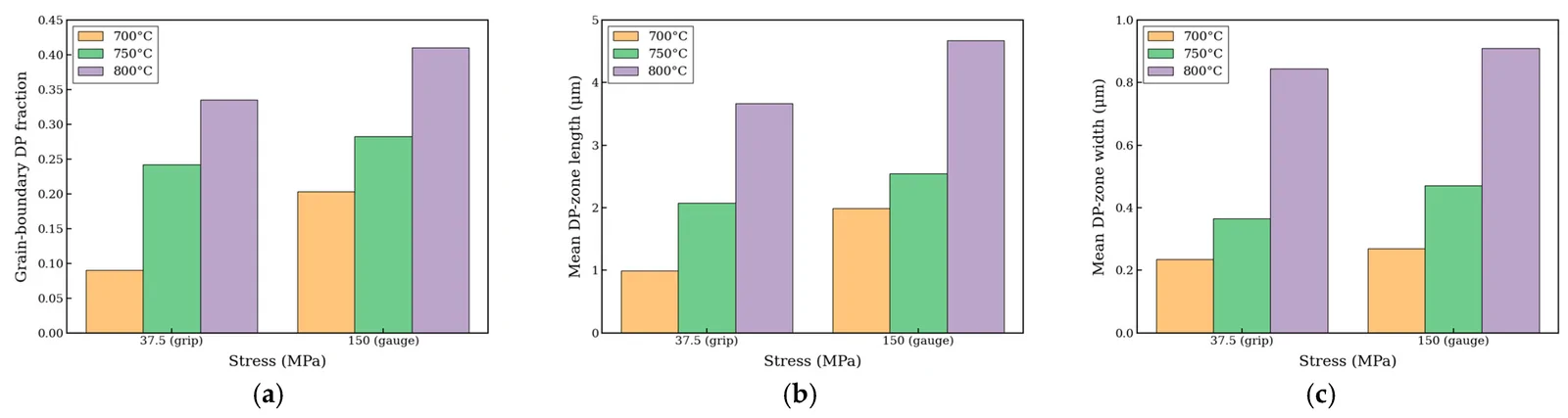
Stress dependence of grain-boundary DP characteristics in the grip and gauge sections at different temperatures: (**a**) grain-boundary DP fraction; (**b**) mean DP-region length; (**c**) mean DP-region width.

To investigate the effect of stress on grain-boundary DP in the Ni–Fe-based alloy, the grain-boundary DP fraction, mean DP-region length, and mean DP-region width were statistically analyzed after 20 h of exposure at 700, 750, and 800 °C under different stress levels. As shown in Figure 22, all three parameters increased with applied stress at a given temperature, demonstrating that external stress promoted the formation and development of grain-boundary DP. The DP fraction was the most stress-sensitive parameter and increased more strongly than the DP-region length and width, indicating that stress primarily promoted the formation and spatial extension of DP regions.

Comparison among the different temperatures further shows that the effect of stress weakened as temperature increased. The differences in the DP parameters caused by stress were most pronounced at 700 °C but became substantially smaller at 800 °C. At high temperature, thermal activation supplied the principal driving force for DP, and stress therefore provided only an auxiliary contribution.

**Figure 23:**
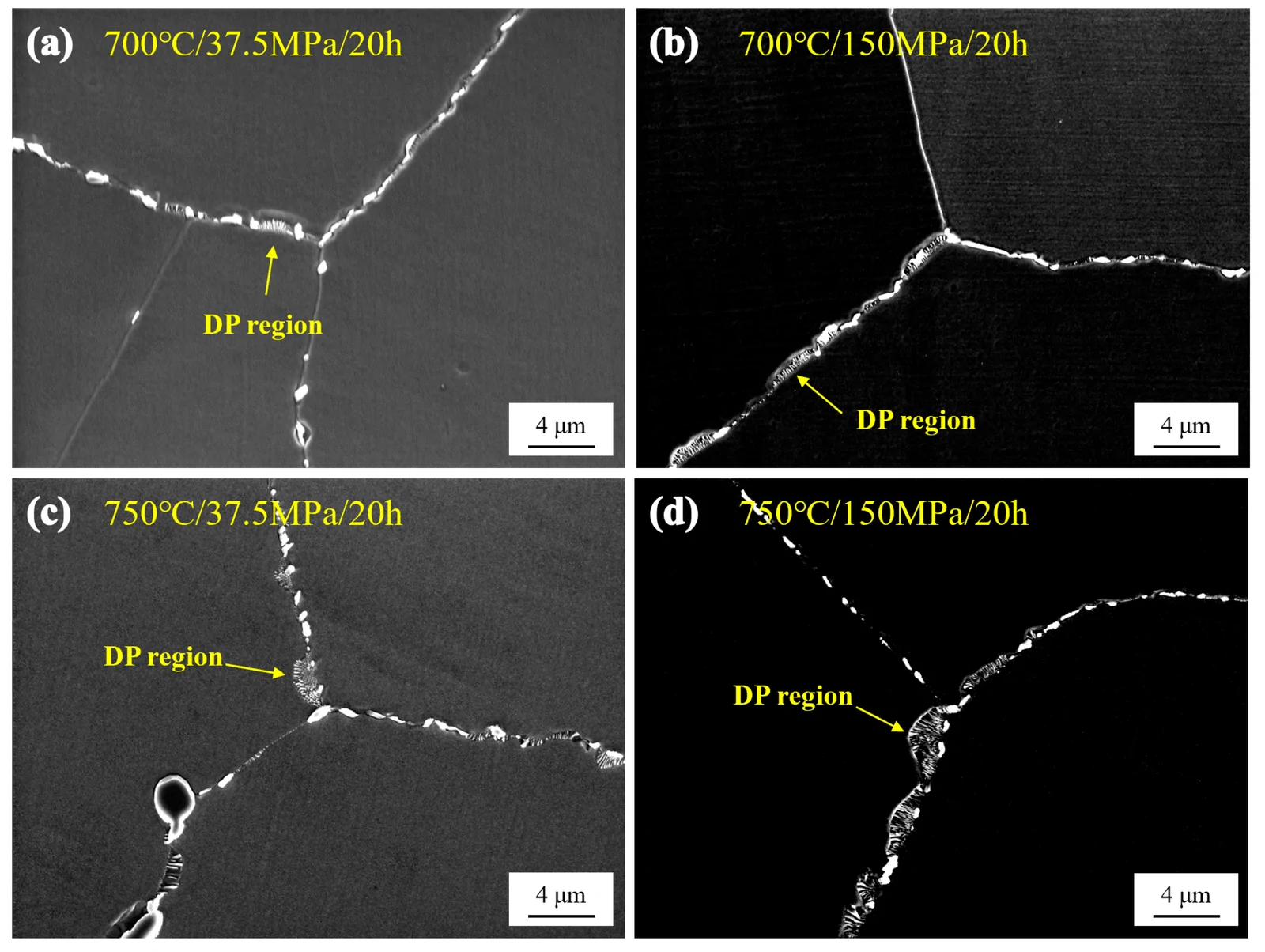
Representative grain-boundary DP regions in Ni–Fe-based alloy at 700 and 750 °C under different stresses.

**Figure 24:**
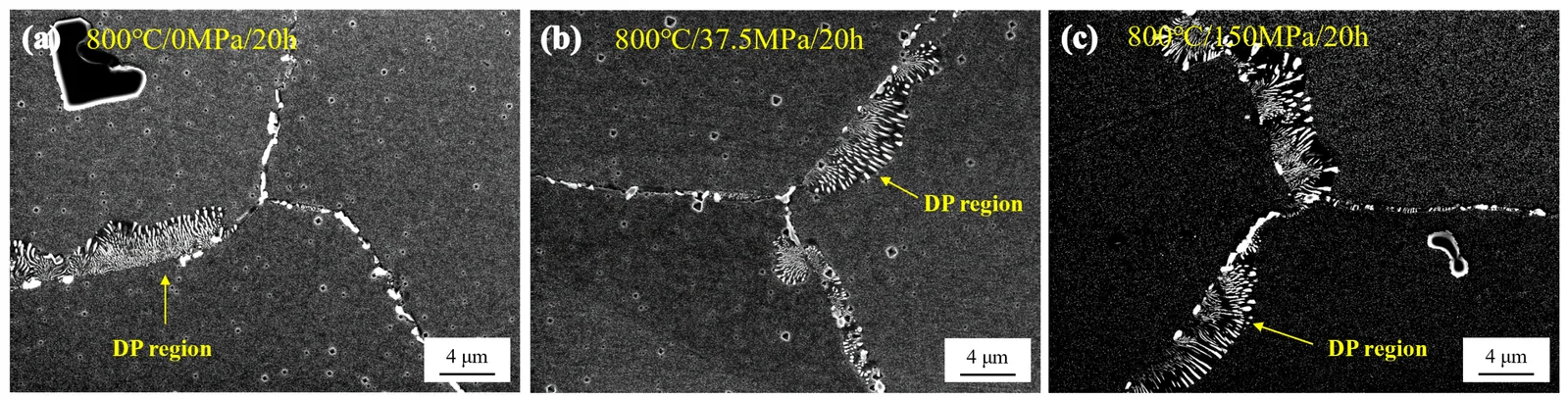
Representative grain-boundary DP regions in Ni–Fe-based alloy at 800 °C under different stresses.

Representative grain-boundary DP morphologies are shown in Figure 23 and Figure 24. At a given temperature, the overall morphology of the DP regions was similar under different stresses, but their number and size increased with applied stress. In particular, no obvious grain-boundary DP was observed in the zero-stress specimen aged at 750 °C, whereas typical DP regions appeared after application of stress at approximately 37.5 MPa, further demonstrating the promoting effect of stress on DP formation.

Overall, stress promoted both the discontinuous precipitation of rod-like γ′ precipitates and the subsequent growth of pre-existing DP regions. Although its effect was weaker than that of temperature, stress remained an important factor promoting grain-boundary DP formation and evolution in the Ni–Fe-based alloy.

#### 3.4.5. Effect of Time on Grain-Boundary DP Evolution

**Figure 25:**
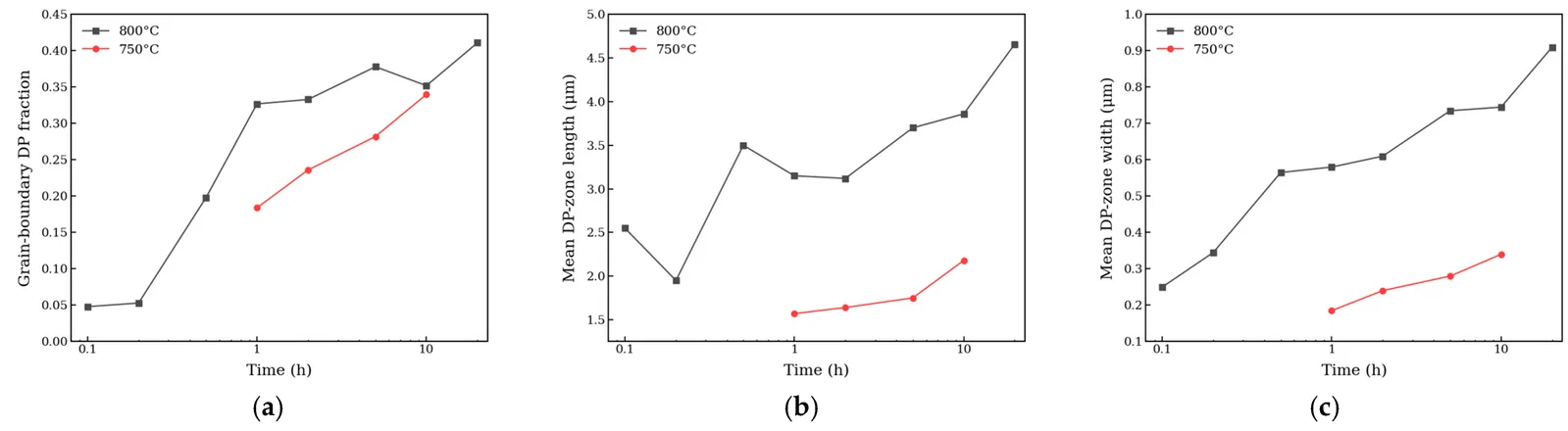
Time dependence of grain-boundary DP characteristics at different temperatures: (**a**) grain-boundary DP fraction; (**b**) mean DP-region length; (**c**) mean DP-region width.

**Figure 26:**
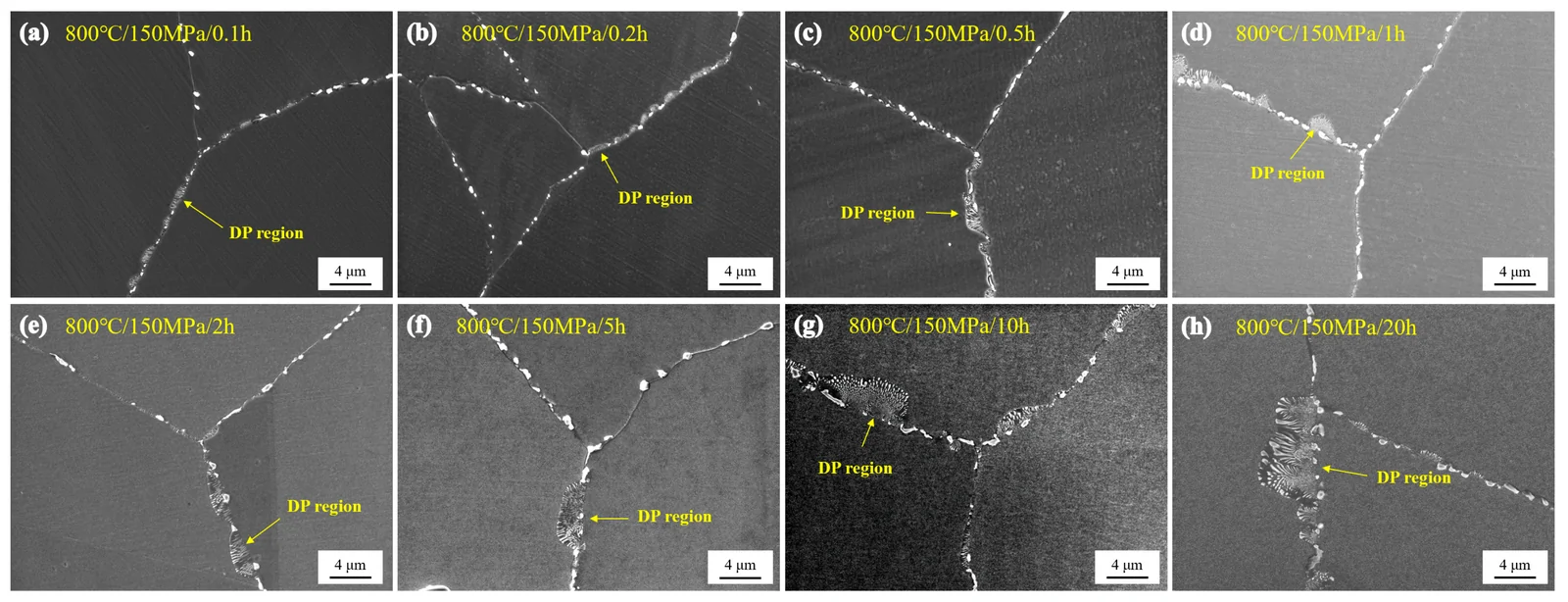
Representative grain-boundary DP morphologies after different exposure times.

To investigate the effect of time on grain-boundary DP in Ni–Fe-based alloys, specimens were exposed at 700, 750, and 800 °C under a stress of 150 MPa for 0.1, 0.2, 0.5, 1, 2, 5, 10, and 20 h. The grain-boundary DP fraction, mean DP-region length, and mean DP-region width were statistically analyzed. The results are shown in Figure 25, and representative DP morphologies are presented in Figure 26.

As shown in Figure 25, the three DP parameters exhibited an overall increasing tendency with exposure time, although local fluctuations were observed. The mean DP-region width showed a pronounced time-dependent increase.

The evolution was distinctly stage-dependent. Overall, DP evolution was more rapid during the early exposure stage and became slower thereafter, although the detailed trends differed among the three characteristic parameters. The reduced DP evolution rate during the later exposure stage is attributed primarily to progressive solute depletion and the resulting decrease in matrix supersaturation. Both intragranular γ′ precipitation and grain-boundary discontinuous γ′ precipitation consume the γ′-forming elements Ti and Al. As DP proceeds, the local availability of these solute elements decreases, reducing the chemical driving force for further precipitation and growth. Consequently, DP formation is rapid during the initial stage but becomes progressively slower at longer exposure times. Although changes in grain-boundary mobility may also contribute to this behavior, this factor was not quantified in the present study. The morphologies in Figure 26 show that numerous rod-like γ′ precipitates formed rapidly during the early exposure period, producing characteristic DP regions. Subsequently, these regions grew and coarsened more slowly through grain-boundary migration and elemental diffusion.

Grain-boundary DP in the Ni–Fe-based alloy therefore followed a two-stage evolution consisting of rapid formation followed by slow growth, rather than a linear process. Temperature and stress primarily governed the formation conditions and evolution rate, whereas time determined the final extent of DP. The initial exposure period was the most active stage for DP formation and microstructural evolution and thus the critical period for the rapid loss of grain-boundary stability.

## 4. Conclusions

(1) All welded-joint specimens fractured in the HAZ or BM on the Ni–Fe-based alloy side, indicating that this side was the creep-critical region under the investigated conditions. Three failure regimes were identified according to the relative creep-rupture performance of the welded joints and Ni–Fe-based alloy BM: Mode I, in which the joint and BM exhibited broadly comparable creep-rupture performance and failure could occur in either the HAZ or BM; Mode II, in which the joint exhibited moderately reduced creep-rupture performance and failure occurred predominantly in the fine-grained HAZ; and Mode III, in which the joint exhibited markedly reduced creep-rupture performance and creep damage was strongly localized in the HAZ, except where a local mixed-grain BM region controlled fracture.
(2) The welded joints exhibited predominantly intergranular creep fracture. Creep cavities and microcracks were preferentially associated with grain-boundary discontinuous precipitation regions containing coarsened rod-like γ′ precipitates and precipitate-free zones, suggesting that DP contributed to grain-boundary weakening and creep-damage accumulation.
(3) The relative extent of DP evolution in the HAZ and BM was closely related to the localization of creep damage and the observed failure regime. A smaller difference in DP extent resulted in comparable damage levels, whereas a larger difference promoted damage localization in the HAZ.
(4) Temperature had the strongest influence on DP formation and growth, while applied stress accelerated DP evolution. DP developed rapidly during the initial exposure stage and subsequently grew at a lower rate, primarily because progressive consumption of γ′-forming solute elements reduced the matrix supersaturation and the chemical driving force for further precipitation.

## Figures and Tables

**Table 1:** Chemical compositions of the base metals and filler wire (wt.%).

Material	Fe	Ni	Cr + Mo + Co	Al + Ti	C	Mn	Cu
Ni–Fe-based alloy	40.0–45.0	Bal.	18.0–21.0	3.0–4.5	0.03–0.08	≤0.3	≤0.05
Inconel 617	2.0–3.0	Bal.	35.0–50.0	1.2–2.1	0.05–0.15	≤1.0	0.2–0.5
Filler wire	3.0	Bal.	45.0	2.0	0.03	1.0	0.5

## Data Availability

The original contributions presented in the study are included in the article, further inquiries can be directed to the corresponding author.
